# Mechanical performance of one part alkali activated concrete incorporating RMC wash water

**DOI:** 10.1038/s41598-026-46730-4

**Published:** 2026-04-06

**Authors:** Hamsashree, Poornachandra Pandit

**Affiliations:** Manipal Institute of Technology, Manipal Academy of Higher Education, Manipal, India

**Keywords:** One-part alkali-activated concrete, Reuse of wastewater, Sustainable materials, Resource efficiency, Carbon dioxide emission, Engineering, Environmental sciences, Materials science

## Abstract

This study investigates the development of one-part alkali-activated (OPAA) concrete using ready-mix concrete (RMC) wash water as a complete replacement for potable water. Fly ash (FA) and ground granulated blast furnace slag (GGBS) were used as primary binders, with the GGBS content fixed at 30%, while the solid activator dosage and water-to-binder (w/b) ratio were varied. The effects of wash water on fresh properties, mechanical performance, durability characteristics, and microstructure were evaluated. Microstructural and mineralogical analyses were conducted using scanning electron microscopy (SEM) and X-ray diffraction (XRD). The OPAA concrete incorporating wash water achieved a maximum compressive strength of 50.67 MPa at 28 days with 14% solid activator and a w/b ratio of 0.32, exceeding the strength of the potable water counterpart of 48.89 MPa. The enhanced performance is attributed to the higher ionic content of wash water, which increased system alkalinity and promoted effective dissolution of aluminosilicate phases. Lower water absorption and sorptivity values indicated a denser microstructure, corroborated by the formation of well-developed C–A–S–H and N–A–S–H gels. Furthermore, OPAA concrete exhibited approximately 70% lower CO_2_ emissions compared to conventional OPC concrete of the same strength grade, highlighting its potential as a sustainable construction material.

**Supplementary Information:**

The online version contains supplementary material available at 10.1038/s41598-026-46730-4.

## Introduction

Water scarcity is one of the defining challenges of the twenty-first century, resulting from a growing imbalance between freshwater demand and limited available resources. Currently, India experiences the world’s largest annual water gap, around 124.3 km^3^ yr^−1^, in total freshwater availability, including surface water and groundwater, relative to regional water demand. Climate projections suggest this deficit could increase by an additional 11.1 km^3^ yr^−1^ under a 1.5 °C warming scenario and by 17.2 km^3^ yr^−1^ under 3 °C, highlighting escalating stress on the country’s freshwater systems^1–3^. Rising water demand driven by population growth, urbanization, pollution, expanded irrigation, and industrial activities further exacerbates this scarcity^4,5^. In this context, improved water management strategies and the adoption of circular economy principles are increasingly recognized as effective approaches for enhancing the efficiency and sustainability of water use^6–8^.

Concrete production requires a substantial amount of water not only for mixing but also for aggregate washing and cleaning of batching equipment and transport trucks. One potential strategy for reducing freshwater consumption in concrete production is the use of alternative water sources, including wastewater, if it satisfies the quality requirements specified in relevant standards. In India, mixing water for concrete must comply with limits prescribed in Indian Standards regarding sulphates, total suspended solids (TSS), alkalinity, total solids (TS), and chlorides. Previous studies have investigated the use of various wastewater sources in concrete production and curing^9,10^. The presence of dissolved solids, organic matter, and suspended particles in wastewater may influence cement hydration and microstructural development, which can affect the mechanical performance of concrete^11^. However, most studies report that the resulting reduction in compressive strength generally remains below 10%, which is within acceptable limits defined by relevant standards^12^.

Among the alternative water sources explored, Ready-Mix Concrete (RMC) wash water has gained increasing attention as a potential replacement for potable water. RMC plants generate significant volumes of wash water during the cleaning of mixers, trucks, and batching equipment, typically ranging from 500 to 1500 gallons per day per plant, creating both environmental and disposal challenges^13,14^. Consequently, several countries have introduced regulations restricting its discharge without treatment. Reusing RMC wash water in concrete production therefore represents a practical approach to reducing freshwater consumption while minimizing environmental impacts^15^. Although several studies have investigated the incorporation of wash water in conventional ordinary Portland cement (OPC) concrete^16–19^, its use in one-part alkali-activated concrete systems remains largely unexplored^20^.

Alkali-activated materials have emerged as promising alternatives to conventional Portland cement due to their ability to utilize industrial by-products such as fly ash and slag as primary binders. When activated with alkaline materials, these systems form aluminosilicate reaction products that can provide high early strength, improved durability, and significantly lower CO_2_ emissions compared with OPC-based systems^21–23^. Alkali-activated binders exhibit fundamentally different reaction mechanisms and microstructural development compared with OPC systems, which may influence how dissolved ions and suspended particles present in wash water interact with the binder matrix. Consequently, the effects of wash water on the workability, strength development, durability-related properties, and microstructure of one-part alkali-activated concrete remain insufficiently understood.

Therefore, this study investigates the feasibility of utilizing RMC wash water as a replacement for potable water in one-part alkali-activated concrete (OPAA) designed for structural applications targeting M40-grade compressive strength at 28 days. The influence of wash water on fresh properties, mechanical performance, durability indicators, and microstructure is evaluated and compared with mixes prepared using potable water. In addition, a comparative assessment of CO_2_ emissions and material costs between OPAA and OPC concrete of equivalent strength grade is conducted to evaluate the environmental and economic implications of the proposed approach.

## Materials

### Binder material

**Fig. 1:**
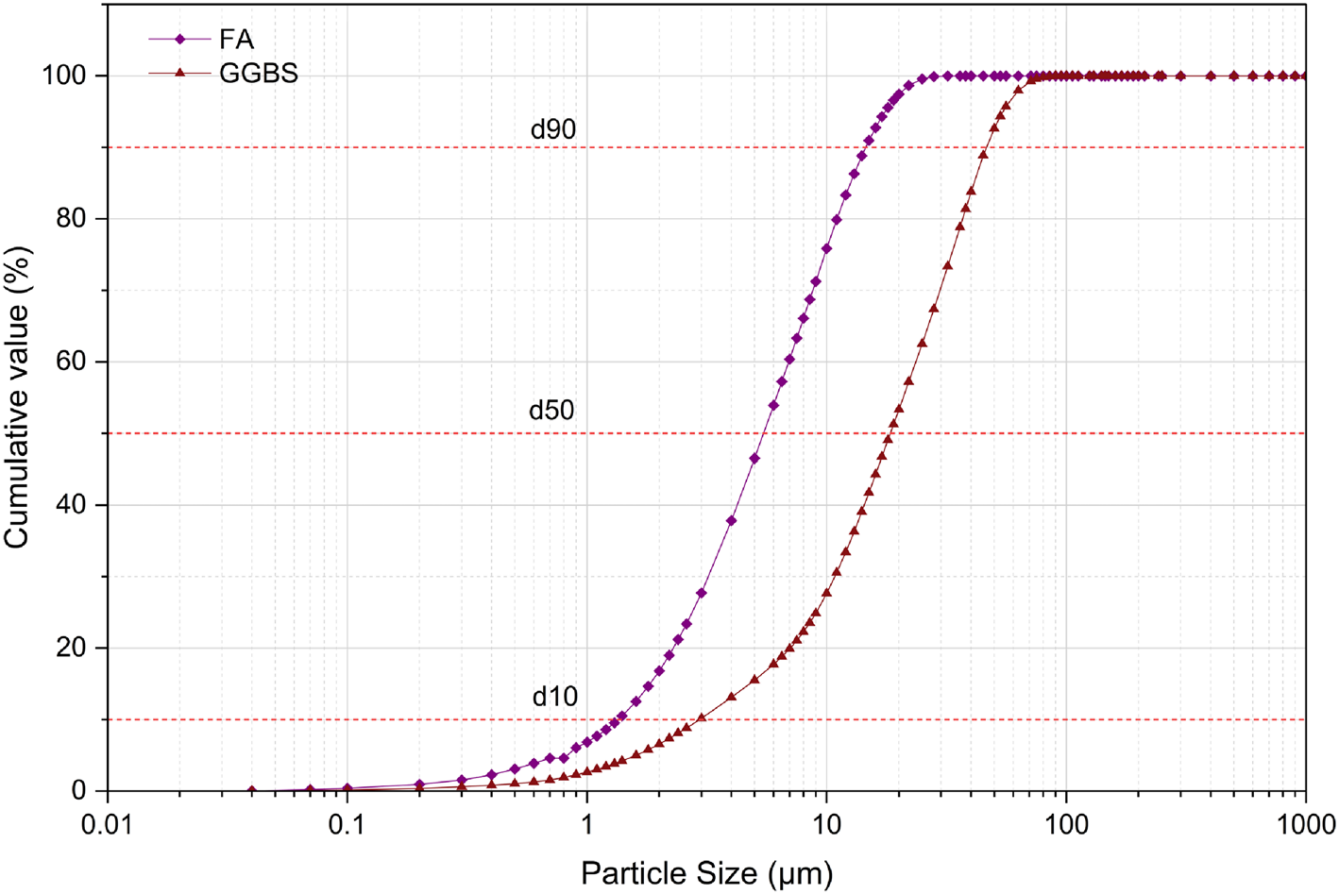
Particle size distribution of binder materials.

**Fig. 2:**
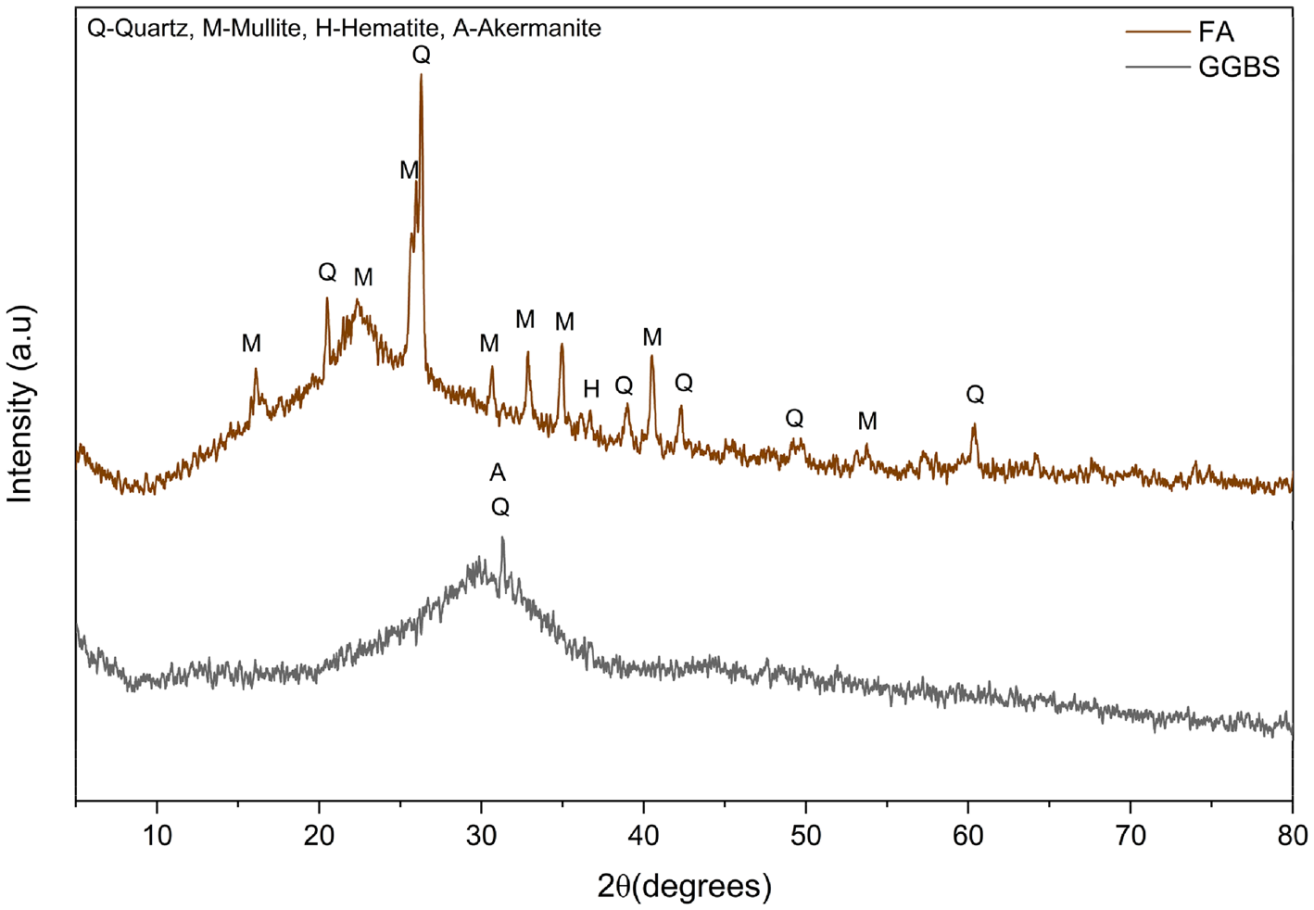
X-ray diffraction of binder materials.

**Fig. 3:**
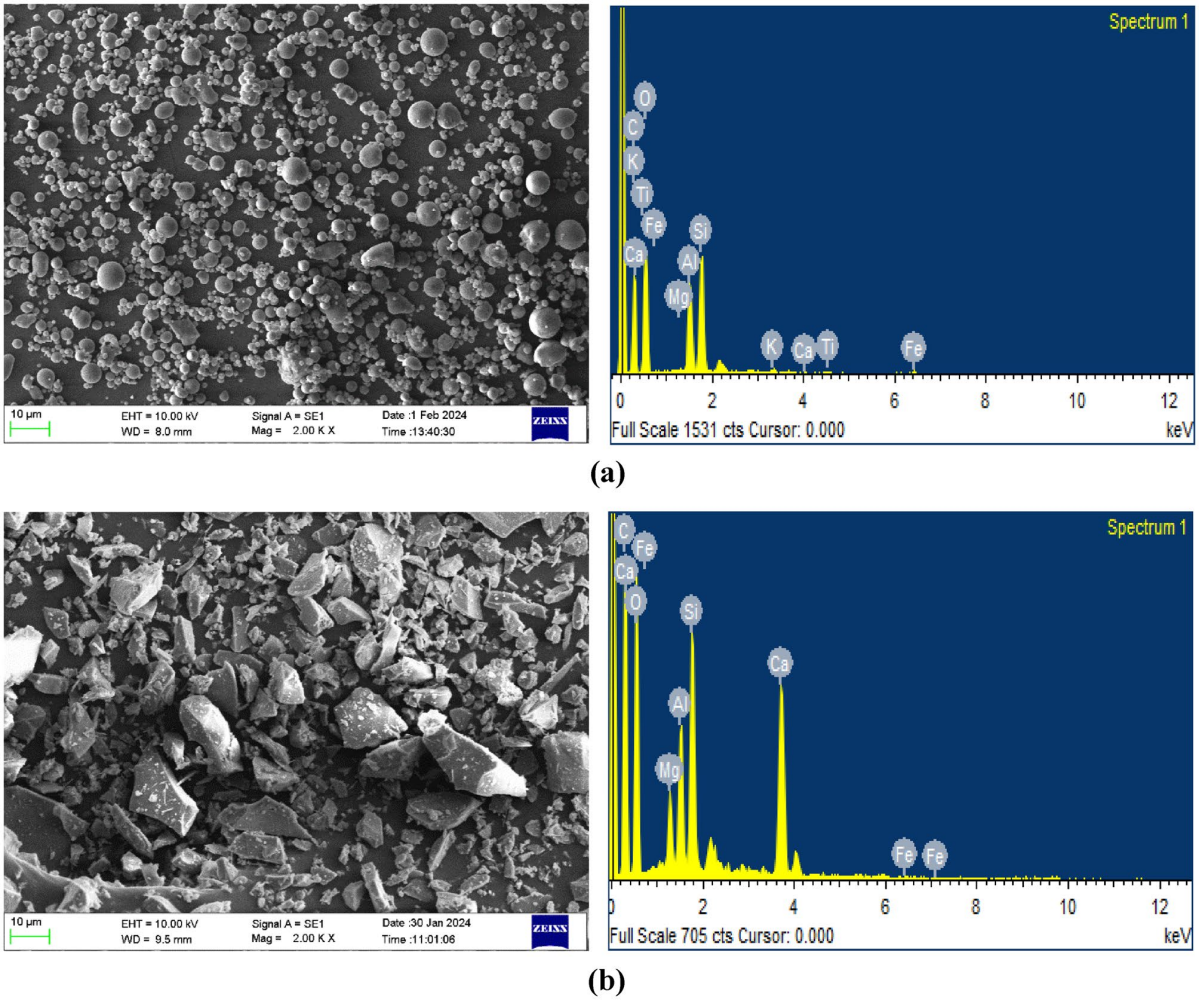
(**a**) SEM–EDS of FA. (**b**) SEM–EDS of GGBS.

The binder used in the preparation of one-part alkali-activated concrete are Fly ash (FA) and ground granulated blast furnace slag (GGBS). The FA was procured from the Bellary Thermal Power Station (Karnataka, India), and the GGBS was sourced from Quality Polytech, Mangaluru, India. Tables 1 and 2 presents the physical and chemical analysis of binder materials respectively. The particle size distribution (PSD) shown in Fig. 1 indicates that FA is considerably finer than GGBS, with FA having a lower mean particle size of 6.87 µm and GGBS exhibiting a coarser mean particle size of 22.31 µm, as presented in Table 1. The chemical composition presented in Table 2 indicates that the fly ash (FA) is classified as low-calcium (Class F) according to ASTM C618, exhibiting high pozzolanic activity, while the ground granulated blast furnace slag (GGBS) is calcium-rich, reflecting its latent hydraulic nature. The X-ray diffraction (XRD) patterns in Fig. 2 illustrate the mineralogical phases present in the binder materials, FA and GGBS. The XRD pattern of FA reveals the presence of both crystalline and amorphous phases, which govern its pozzolanic reactivity. In contrast, GGBS exhibits a predominantly amorphous phase, reflecting its highly reactive glassy structure by a broad diffuse hump cantered around 2θ ≈ 29–32°. Surface morphology and elemental composition of the binders were examined using Scanning Electron Microscopy coupled with Energy-Dispersive X-ray Spectroscopy (SEM–EDS). Figure 3a shows FA which consists mainly of smooth, spherical glassy particles, with EDS spectra showing dominant peaks of Si, Al, and O confirming its silica–alumina–rich, pozzolanic nature. In contrast, Fig. 3b shows GGBS particles with an irregular, angular morphology and pronounced EDS peaks for Ca, Si, Al, and O, along with traces of Mg, indicating its calcium-rich amorphous matrix.

**Table 1:** Physical properties of binder materials.

Material	FA	GGBS
Specific gravity	2.48	2.91
Specific surface area (m^2^/g) (BET method)	1.73	0.59
Particle size (µm)		
d10	1.34	2.94
d50	5.45	18.43
d90	14.54	46.44
Mean Particle Size (µm)	6.87	22.31

**Table 2:** Chemical properties of binder materials (% by mass).

Chemical oxides	SiO_2_	Al_2_O_3_	Fe_2_O_3_	TiO_2_	K_2_O	CaO	P_2_O_5_	SO_3_	MgO	MnO	Loss of ignition
FA	55.8	28.2	7.45	2.92	2.05	1.54	0.532	0.495	0.44	0.069	1.1
GGBS	30.1	15.4	0.585	1.16	0.498	42.9	ND	1.3	6.17	1.57	0.32

*ND* not detected.

### Activators

The solid alkaline activators used in this study was sodium hydroxide flakes (purity greater than 99%) and sodium metasilicate pentahydrate powder supplied by Astra chemicals, Chennai, India. The physical and chemical properties of sodium hydroxide and sodium silicate are presented in Tables 3 and 4 respectively.

**Table 3:** Physical and chemical properties of Sodium hydroxide.

Properties	Sodium hydroxide
Molecular formula	NaOH
Molar mass	39.9463 g/mol
Physical state/appearance	White flake
Solubility in water	111 g/100 ml (20° Celsius)
Specific gravity	3.10

**Table 4:** Physical and chemical properties of Sodium silicate.

Properties	Results
Molecular formula	Na_2_SiO_3_⸱5 H_2_O
Molar mass	122.01 g/mol
Physical state/appearance	White powder
Specific gravity	2.61
Wt. Ratio Na_2_O/SiO_2_	1:1 + 0.05
Na_2_O	29% by weight
SiO_2_	29% by weight
Water	43.5%
pH	12.8

### Aggregates

**Fig. 4:**
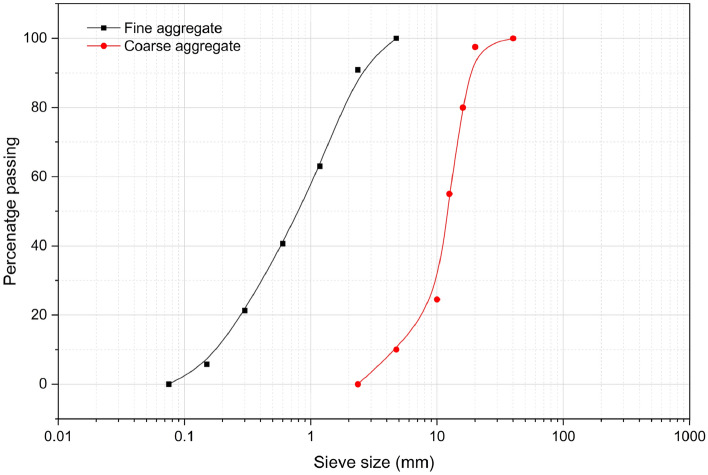
Particle size distribution curve of aggregates.

The natural coarse aggregate and fine aggregate (M-sand: Zone-II) used in the study was procured from Udupi, Karnataka confirming the specification of IS:383 (Part 1)-2016^24^. The particle size distribution of fine and coarse aggregates is presented in Fig. 4. Tests on aggregates were performed based on IS: 2386–1963^25^ and are detailed in Table 5.

**Table 5:** Physical properties of aggregates.

Properties	Fine aggregate	Coarse aggregate
Specific gravity	2.60	2.65
Fineness modulus	3.20	7.10
Bulk density (loose state) (kg/m^3^)	1548	1428
Bulk density (compacted state) (kg/m^3^)	1698	1586
Water absorption (%)	1.02	0.45
Bulking of sand (%)	26	–

### Water

The wastewater used in this study was ready-mix concrete (RMC) wash water collected from the settlement tank of RMC Prism, Manipal, in April 2023, one day after truck cleaning. Wash water was collected from a single ready-mix concrete plant, and the same batch of wash water was used throughout the experimental programme. It was stored in sealed containers under laboratory conditions until use. To verify the stability of its composition, the physicochemical characteristics of the wash water were analysed twice during the experimental period. The results indicated only minor variation in the measured parameters, confirming that the wash water composition remained reasonably consistent for the experimental programme. Potable water was obtained from the local municipal supply. Chemical analysis in Table 6 showed higher concentrations of solids, chloride, sulfate, and alkalinity in wash water, though within permissible limits of IS 456:2000^26^ and ASTM C1602:2018^27^. ICP-OES results in Table 7 showed elevated levels of Ca^2+^, Na^+^, and K^+^ ions in wash water, contributing to its higher alkalinity and pH, which can influence reactivity, durability, and binder performance in alkali-activated systems.

**Table 6:** Properties of potable water and wash water.

Parameter	Potable water	Wash water	IS 456: 2000 permissible limit	ASTM C1602:2018 permissible limit
Chloride (mg/L)	9.93	77	500^b^	500^a^
			2000^c^	1000^b^
Sulphate (mg/L)	53.45	156	400	3000
Total solids (mg/L)	100	1165	2000	50,000
pH	6.9	11.73	≥ 6	–
Alkalinity (mg/l)	60	460	–	600

^a^In prestressed cement concrete, ^b^In reinforced cement concrete, ^c^In plain cement concrete.

**Table 7:** Ionic composition of water samples (in mg/L).

Ion’s mg/l	Potable water	Wash water
Ca	18.411	115.4
Na	6.559	90.305
K	2.953	123.283
Al	0	1.422
Fe	0.07	0.021
Mg	17.089	51.5
Sr	0.04	1.33
Zn	0.361	0.006
Mn	0.016	0.163
Li	0.02	0.294
Cu	0.011	0.005
Cr	0.004	0.071
Ba	0	0.056

## Experimental methodology

### Design mixtures and specimen preparation

M40 grade concrete was selected to satisfy the strength and durability requirements for high-performance structural applications, in accordance with IS 456:2000 provisions for extreme exposure. While IS 456:2000 does not specify mix design procedures for alkali-activated concrete (OPAAC), its guidelines on water quality and aggregates remain relevant. IS 17452:2020^28^ provides recommendations for two-part alkali-activated concrete in non-structural products but excludes one-part systems. Therefore, a systematic experimental optimisation programme was undertaken to develop the mix proportions. The mix design was guided by the volumetric principles of IS 10262:2009^29^ and relevant literature to achieve the target strength and workability. A total of eight preliminary mixes were prepared during the optimisation stage to evaluate the influence of FA/GGBS ratio, activator dosage, and water-to-binder ratio on workability and 28-day compressive strength. The final mix proportions were selected based on these results, and the details of the preliminary mixes are provided in the Supplementary Material, Table S1. Although higher slag contents (30–35%) exhibited increased compressive strength, 25% GGBS was adopted as a balanced proportion, as it was expected to achieve the target M40 strength while maintaining workable consistency and stable reaction behaviour under ambient curing.

Based on the findings of the preliminary optimisation study, the final mix proportions were selected for detailed investigation in the present study. Accordingly, a total of 12 mixes were prepared with the constant binder content and aggregate volume fractions in saturated surface-dry condition. The quantities of binder, aggregates, water, and activator derived from the calculations are presented in Table 8. The quantities of all constituents were calculated for a unit concrete volume of 1 m^3^, allowing consistent and scalable mix proportions for all mixtures. The water quantities were maintained constant for all activator dosages within each w/b ratio (0.32 and 0.34). Depending on the mix designation, either potable water (PW) or wash water (WW) was used. Mix notations follow the convention in Table 9, e.g., ‘32P10’ represents w/b = 0.32, potable water, and 10% activator and similar notation applies to other group numbers.

**Table 8:** Mix proportions for M40 grade one-part alkali-activated concrete per cubic meter.

Binder kg (FA: GGBS = 75:25)	Fine kg aggregates	Coarse kg aggregates	Activator kg (SH: SS = 1:2)	Water kg
450	614	1129	45 (10%) 54 (12%) 63 (14%)	144 (w/b-0.32) 153 (w/b-0.34)

SS- Sodium Silicate; SH- Sodium Hydroxide.

**Table 9:** Mix proportions and variables used in the OPAA concrete mixes.

Mix ID	FA/GGBS (%)	Water	w/b ratio	Activator (%)
32P10	75/25	PW	0.32	10
32P12				12
32P14				14
34P10	75/25	PW	0.34	10
34P12				12
34P14				14
32W10	75/25	WW	0.32	10
32W12				12
32W14				14
34W10	75/25	WW	0.34	10
34W12				12
34W14				14

*PW* potable water, *WW* wash water, *w/b* water to binder ratio.

**Fig. 5:**
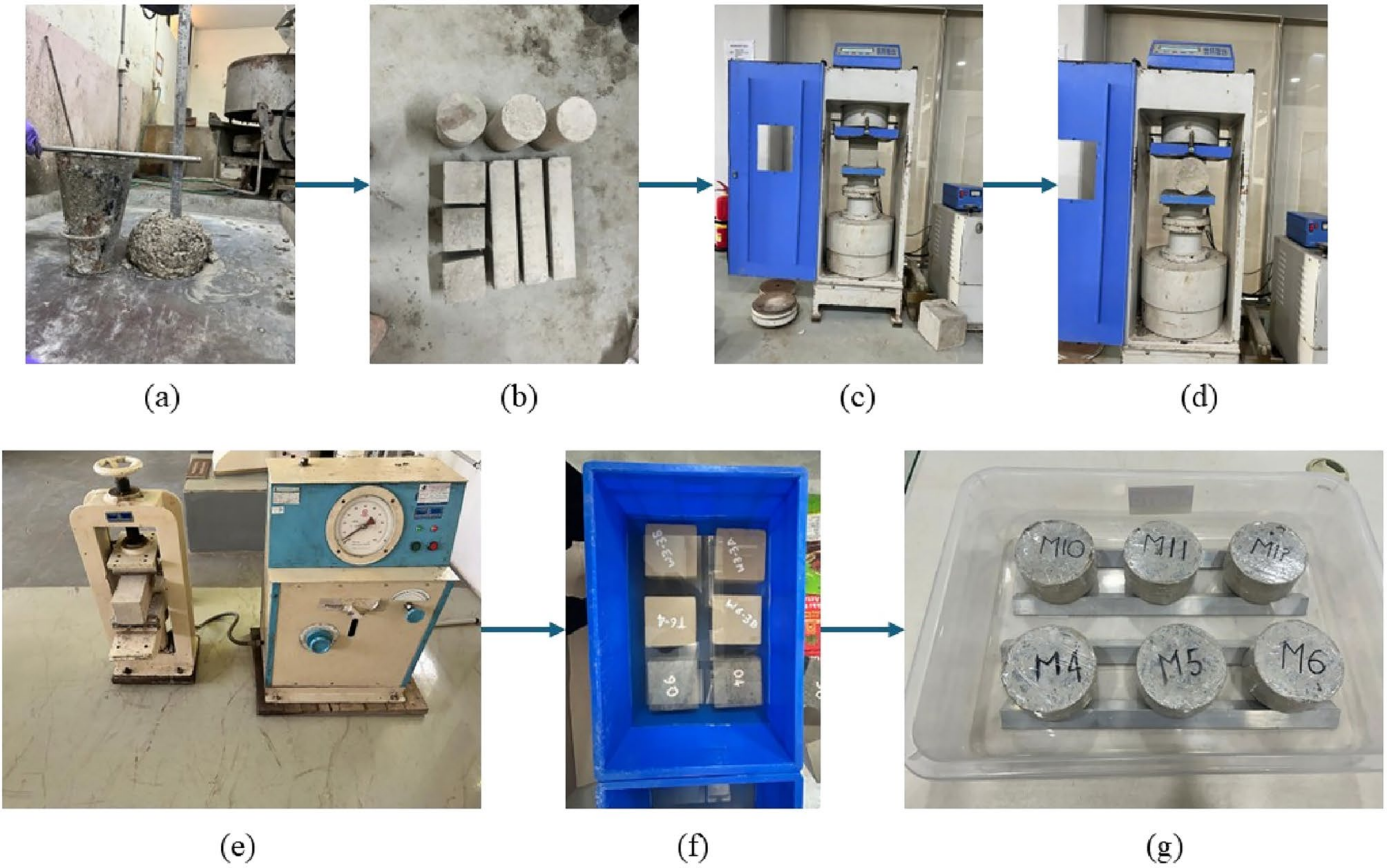
Experimental test setups used in this study. (**a**) Slump test, (**b**) Ambient curing, (**c**) Compression test, (**d**) Split tensile test, (**e**) Flexural strength test, (**f**) Water absorption, (**g**) Sorptivity

All mixes were prepared using a vertical mixer. Fine and coarse aggregates in a saturated surface-dry condition were first mixed for 1 min, followed by FA and GGBS for another minute. The activator was added and mixed for 2 min, after which water was gradually introduced and mixing continued for 3 min to ensure uniformity. The fresh concrete was tested for workability, then cast in moulds in three layers and compacted on a vibrating table. Specimens were demoulded after 24 h and ambient cured at 27 °C and 75 ± 10% relative humidity until testing as shown in Fig. 5b.

An M40-grade OPC concrete was prepared as the control mix to provide a reference for strength comparison. The mix proportions are provided in the Supplementary Material. The mixing, casting, and compaction procedures were identical to those adopted for the alkali-activated concrete mixes. After demoulding at 24 h, the OPC specimens were subjected to standard water curing until the age of testing.

### Test methods

After mixing, the fresh and hardened properties of one-part alkali-activated concrete (OPAAC) were evaluated. Slump tests were conducted as per IS 1199 (Part 2): 2018^30^ to assess workability. Ultrasonic pulse velocity (UPV) tests were performed at different curing ages to examine internal homogeneity as per IS 516 (Part 5/Sec 1): 2018^31^. Mechanical tests—including compressive, split tensile, and flexural strength—were conducted at 7, 28, 56, and 90 days as per IS 516 (Part 1/ Sec 1): 2018^32^. A 2000 kN universal testing machine was used, applying loading rates of 2.4 kN/s and 0.05 kN/s for compressive and flexural tests, respectively. The average of three specimens per mix was reported for mechanical properties. Durability tests such as water absorption and sorptivity, were performed at 28 and 56 days as per ASTM C642-21^33^ and ASTM C1585-13^34^ respectively with two specimens per mix. The test details and corresponding standards are summarized in Table 10, and setups are shown in Fig. 5.

**Table 10:** Experimental test parameters and specimen details.

Tests performed	Specimen size mm	Curing ages days	Number of specimens	References
Slump	Fresh concrete	–	–	IS 1199 (Part 2): 2018^30^
Ultrasonic pulse velocity	150 × 150 × 150 Cube	28/56/90	117	IS 516 (Part 5/Sec1): 2018^31^
Compressive strength	150 × 150 × 150 Cube	7/28/56/90	156	IS 516: 2018^32^
Split tensile strength	150 D × 300 H Cylinder	7/28/56/90	156	IS 516:2018
Flexural strength	100 × 100 × 500 Beam	7/28/56/90	156	IS 516:2018
Water absorption	150 × 150 × 150 Cube	28/56	52	ASTM C642-21^33^
Sorptivity	100 D × 50 H Disk	28/56	52	ASTM C1585-13^34^
SEM–EDS	Small pieces	7/28/56	–	–
XRD	Powder < 75 µm	7/28/56	–	–

*D* diameter, *H* height.

Mixes that achieved the target M40 grade strength, along with those exhibiting lower strength, were selected for microstructural analysis. The mineral phase composition was analyzed using X-ray diffraction (XRD) over a 2θ range of 5°–90° with a step size of 0. 02°.The microstructure and elemental composition were examined using scanning electron microscopy (SEM) and energy dispersive X-ray spectroscopy (EDS), respectively. The XRD scans were performed with a 2θ scanning range of 5° to 90°, and a step size of 0.02°.

## Results and discussions

### Workability

**Fig. 6:**
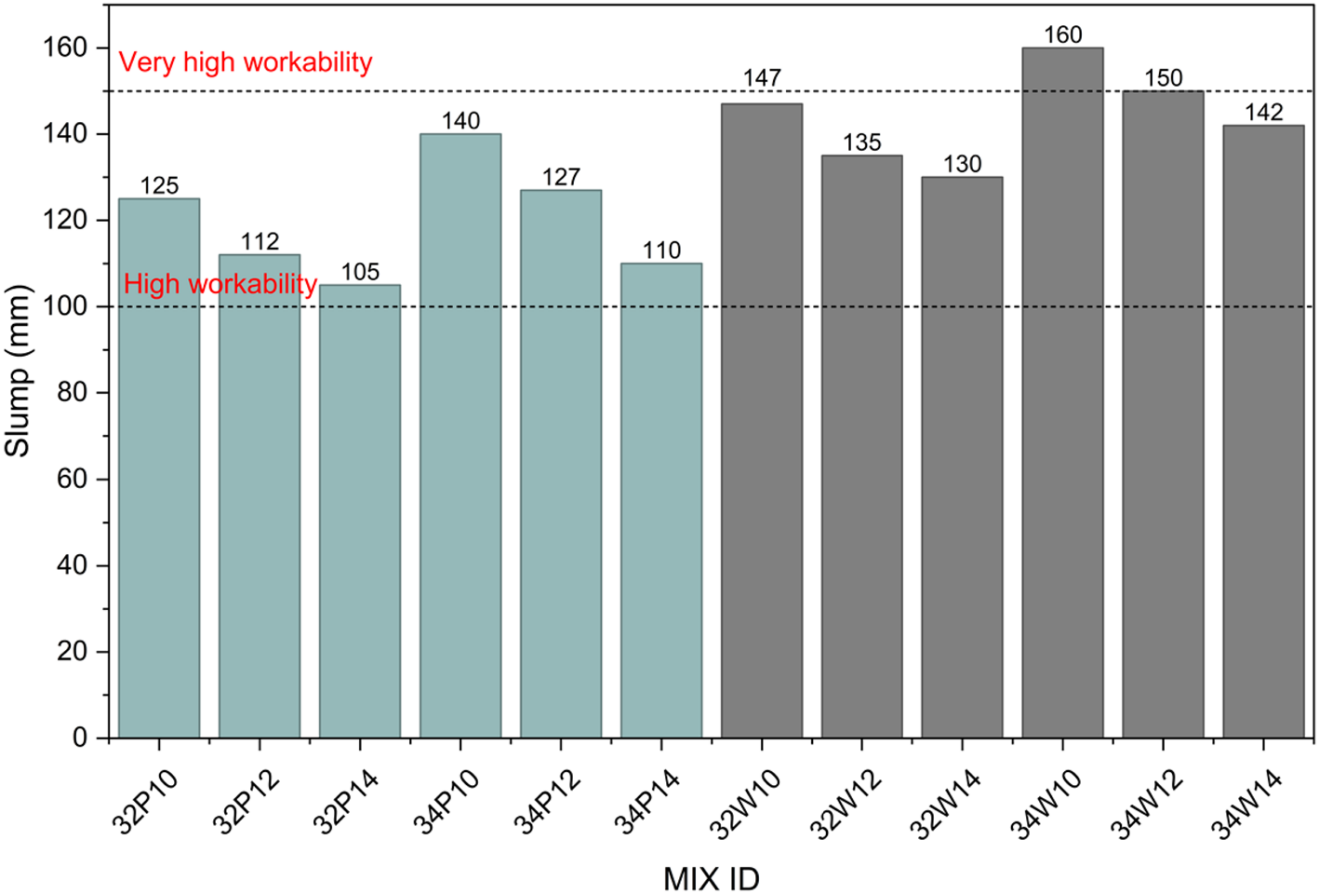
Workability of fresh OPAAC.

The workability of the mixes, measured through slump values was found to be within the recommended range of IS 456:2000 (75–150 mm for RCC and pumped concrete), confirming satisfactory placement characteristics. Slump values represent the mean of two replicate measurements (n = 2), and variability is expressed as standard deviation, as shown by the error bars in Fig. 6. Among the parameters studied, the mixing water had a more pronounced effect on the workability than the variations in activator content and water-to-binder ratio. As shown in Fig. 6, potable water mixes exhibited slumps between 105 and 140 mm, whereas wash water mixes achieved slightly higher values, ranging from 130 to 160 mm. The increase in slump ranged from 14.29% to 29.09%, shows a clear improvement when wash water was used. This improved workability is due to the higher concentration of dissolved ions such as Na^+^, K^+^, Ca^2+^, and Mg^2+^ in the wash water. These ions reduce the repulsive forces between aluminosilicate particles by neutralizing their surface charges, allowing particles to move closer and flow more easily^35^. This effect is related to a lower zeta potential and a thinner electrical double layer around the particles, which reduces internal friction in the mix^36^. Furthermore, the slump values were found to increase with a higher w/b ratio, as higher water content acted as a lubricant, reducing internal friction and enhancing workability^37^. At a constant w/b ratio, an increase in activator content from 10 to 14% consistently reduced the slump values. This reduction is attributed to the higher alkali concentration accelerating reaction kinetics, which promotes rapid setting and water loss due to exothermic reactions, thereby significantly reducing workability, consistent with trends reported in previous studies^38,39^.

### Compressive strength

**Fig. 7:**
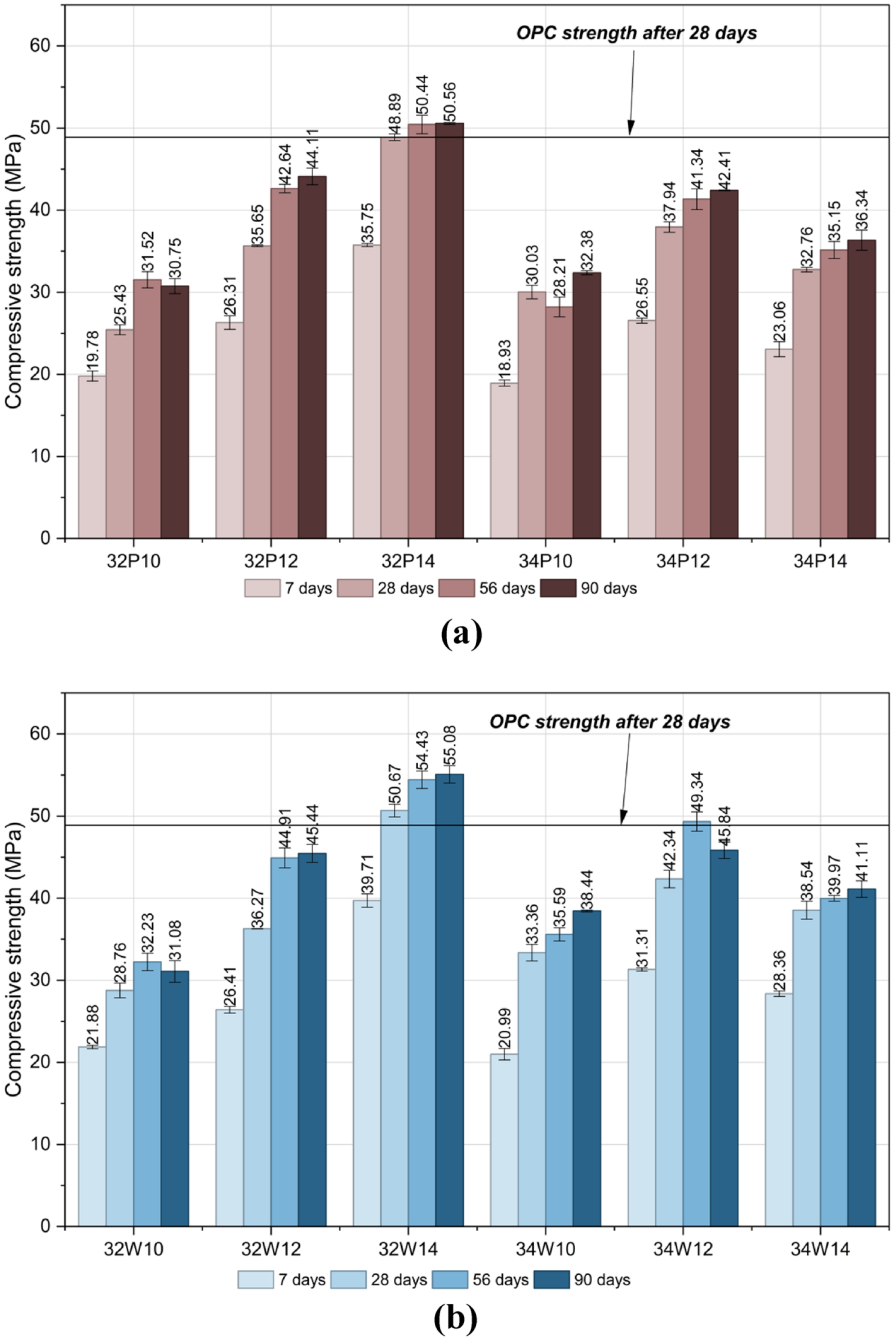
(**a**) Compressive strength of Potable water mixes at different curing ages. (**b**) Compressive strength of Wash water mixes at different curing ages.

All mechanical strength values represent the average of three specimens tested under identical conditions (n = 3). Data variability was expressed in terms of standard deviation and presented as error bars. The individual specimen values used to compute the average strength and standard deviation are provided in Table S2-S4 in Supplementary Material. Figure 7a shows the compressive strength development of potable water mixes at different curing ages. A clear trend is observed where increasing activator content from 10 to 14% enhances strength at all curing ages, owing to improved dissolution of binder particles. The finding is well correlated with that of^40,41^. The compressive strength of mixes with a lower w/b of 0.32 was greater than those with a higher w/b of 0.34. With constant activator content, the compressive strength decreases as the w/b ratio increases, indicating that a w/b ratio of 0.32 yields optimal performance for the OPAAC. An excess of water reduces the mixture’s alkalinity, obstructing the hydroxylation of FA, which subsequently diminishes the reaction extent. Strength gain was progressive with curing age, being most significant between 7 and 28 days, followed by gradual stabilization until 90 days. Among the potable water mixes, 32P14 achieved the highest 28-day compressive strength of 48.9 ± 0.36 MPa, demonstrating strength equivalence with the reference control mix.

To statistically validate these observations, a two-way ANOVA was performed at a 95% confidence level for the strength results at all curing ages. The detailed ANOVA results for the 28-day strength data are presented in Table S5 (Supplementary Material), as this curing age is widely considered the standard reference for evaluating the structural performance of concrete. The results indicate that both w/b ratio (F = 270.41, *p* < 0.0001) and activator dosage (F = 1679.63, *p* < 0.0001) significantly influence compressive strength. The higher F-value associated with activator content demonstrates that activator dosage exerts a more dominant effect compared to w/b ratio. Furthermore, the interaction between w/b and activator was statistically significant (F = 1182.07, *p* < 0.0001), confirming that the influence of activator dosage varies depending on the selected w/b ratio. These statistical findings substantiate the experimentally observed strength trends.

Figure 7b shows the compressive strength of mixes prepared using wash water as the mixing medium. Wash water mixes followed the same strength development trend as potable water mixes with respect to activator content, water-to-binder ratio, and curing age. Strength increased with higher activator concentrations (10 → 12 → 14%), lower w/b ratio (0.32 over 0.34), and longer curing periods. The reduction in compressive strength with increasing w/b ratio can be attributed to the higher amount of free water in the mix, which increases pore volume and reduces matrix density after evaporation. In alkali-activated systems, excess water dilutes the alkaline activator concentration and slows the geopolymerization reaction, leading to a less compact aluminosilicate gel structure and consequently lower compressive strength. At w/b = 0.32 with 14% activator content (32W14), the mix achieved 50.7 ± 0.44 MPa at 28 days, exceeding the OPC control strength (48.9 MPa) by 3.7%.

To statistically validate these observations, a two-way ANOVA was conducted at a 95% confidence level for the strength results at all curing ages. The 28-day strength results are presented in Table S6 (Supplementary Material). The analysis showed that activator dosage significantly influenced compressive strength (F = 865.63, *p* < 0.0001). However, unlike potable water mixes, the effect of w/b ratio was not statistically significant (F = 3.83, *p* = 0.074), indicating that wash water mixes are less sensitive to changes in w/b ratio at 28 days. The significant interaction effect (*p* < 0.0001) suggests that the influence of w/b becomes relevant primarily at specific activator levels.

**Fig. 8:**
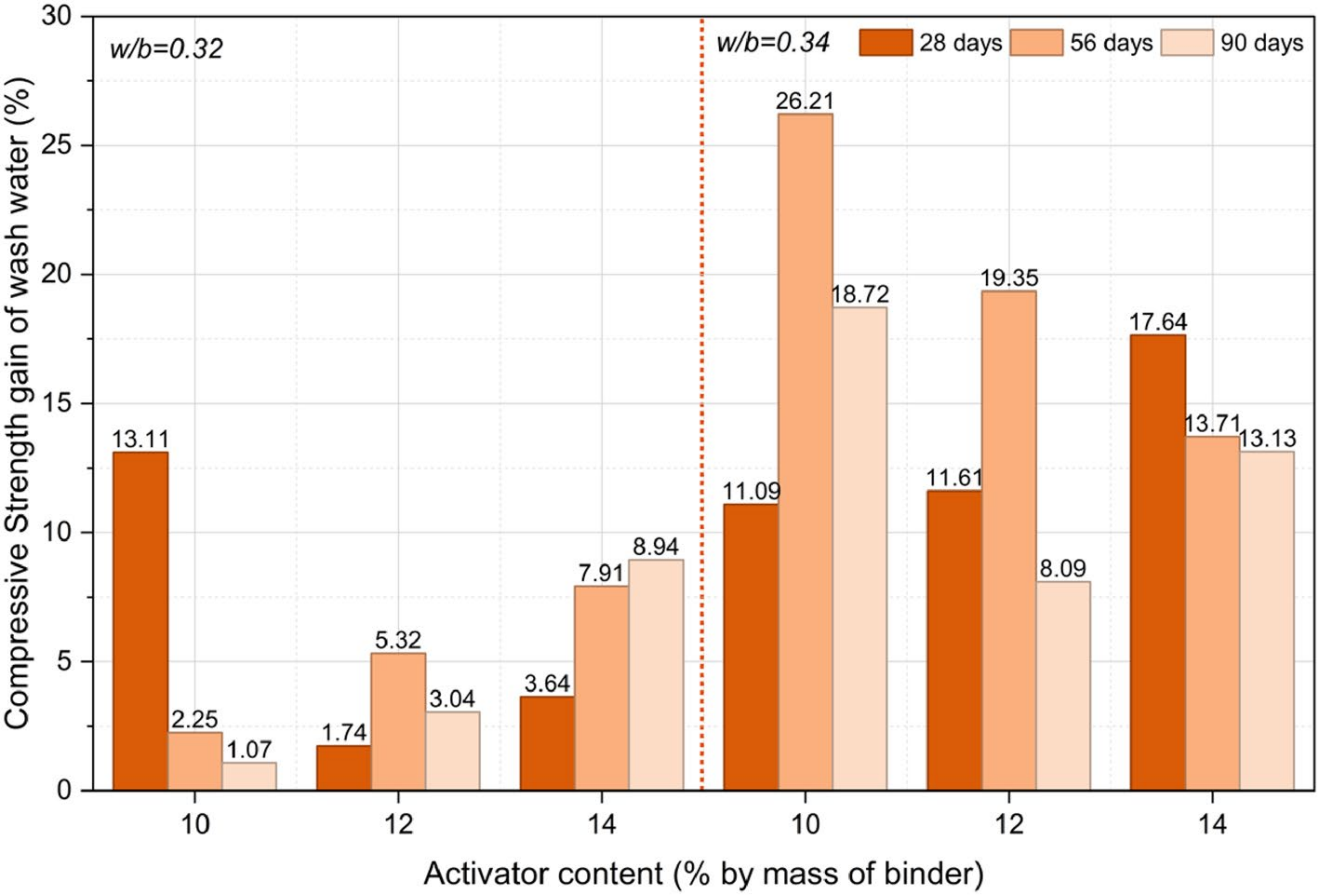
Percentage Strength Gain of Wash water over Potable water at 28, 56 and 90 days.

Figure 8 illustrates the percentage gain in compressive strength of concrete prepared with wash water compared over potable water, evaluated at 28, 56, and 90 days. It should be noted that a higher percentage gain does not necessarily indicate superior absolute performance; rather, it reflects the relative difference with respect to the potable-water baseline, which itself varies with w/b ratio and activator content. The chemical analysis indicates that wash water has higher concentrations of ionic species compared to potable water, increasing the ionic strength and alkalinity of the wash water^42,43^. This elevated alkalinity and ion concentration are expected to enhance hydration kinetics and promote early strength development, consistent with the observed compressive strength results^20^.

Although wash water mixes exhibited consistent numerical strength gains, statistical analysis revealed that the overall effect of water type on 28-day compressive strength was not significant (*p* > 0.05). To further assess the combined influence of water type and activator dosage, a two-way ANOVA was conducted at a 95% confidence level (Table S7, Supplementary Material). The results confirm that water type (potable vs wash water) did not significantly affect compressive strength (F = 3.72, *p* = 0.0632), whereas activator dosage exerted a statistically significant influence (F = 22.16, *p* < 0.0001). The absence of a significant interaction effect (*p* = 0.9467) indicates that the influence of activator dosage is independent of the mixing water type. Collectively, these findings demonstrate that wash water performs comparably to potable water across the investigated activator levels.

*Effect of water-to-binder ration on strength gain of wash water:* Although wash-water mixes showed a higher percentage strength gain when the w/b ratio increased from 0.32 to 0.34, this is mainly attributed to the lower absolute strength of the potable-water reference at w/b = 0.34, which inflates the relative difference rather than indicating enhanced wash-water performance. In absolute terms, mixes with w/b = 0.32 consistently achieved higher compressive strengths at all curing ages for both water types. Although the overall effect of w/b ratio on 28-day strength was not statistically significant in wash-water systems, the observed relative gain may be associated with the elevated ionic content of wash water.

*Effect of activator content on strength gain of wash water*: Activator dosage exerted a statistically significant influence on compressive strength (*p* < 0.0001). For w/b = 0.32, increasing activator content reduced percentage strength gain at 28 days, suggesting that higher alkalinity may have accelerated early reactions and limited further strength enhancement. However, at 56 and 90 days, strength gain improved with increasing activator content, likely due to continued formation of C–A–S–H and N–A–S–H type gels. In contrast, at w/b = 0.34, strength gain increased with activator content at 28 days, reflecting enhanced early-age dissolution. At later ages, the trend reversed, possibly due to excess alkalis and higher pore solution concentration affecting matrix densification.

*Effect of curing age on strength gain of wash water:* For the mix with w/b of 0.32, strength gain exhibited a non-linear trend with curing age. At 10% activator, strength decreased over time, indicating insufficient alkalinity to sustain long-term hydration reactions. With 12% activator, strength increased up to 56 days before stabilizing, suggesting that major reaction products had formed by then. At 14% activator, strength gain continued throughout curing, as higher alkalinity enhanced precursor dissolution and prolonged gel formation. For the mix with w/b of 0.34, the trend differed. At 10% and 12% activator, strength increased up to 56 days but slightly declined at 90 days, likely due to partial densification. At 14% activator, strength consistently decreased with age, implying that excessive alkalinity and higher water content. These observations highlight the coupled influence of activator concentration, curing duration, and ionic composition on strength evolution in wash-water-based systems.

**Fig. 9:**
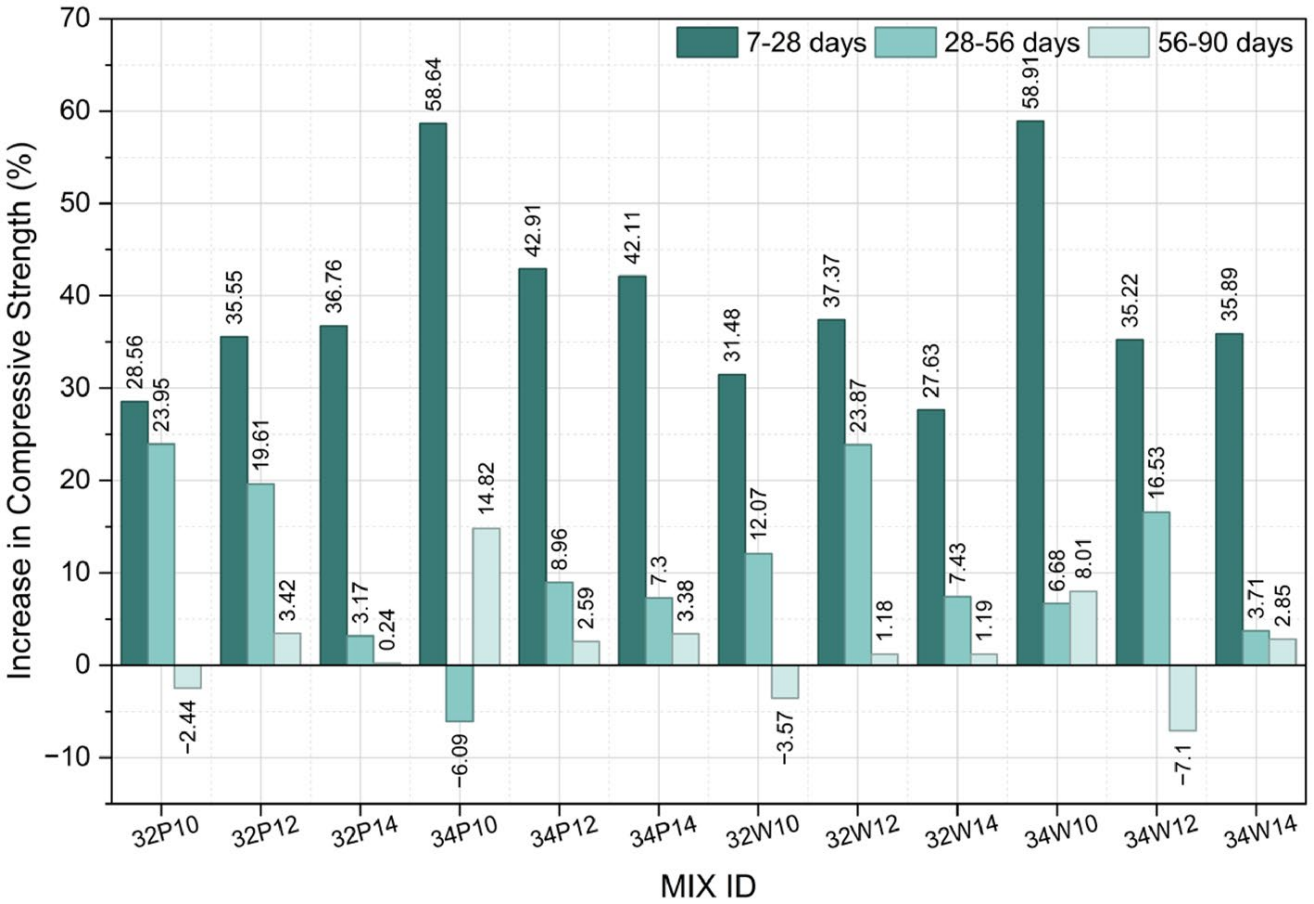
Percentage Strength Gain of OPAAC Mixes Between Successive Curing Ages.

Figure 9 presents the percentage strength gain of all mixes across successive curing intervals. For potable-water mixes, the strength gain from 7 to 28 days ranged from 28.6 to 58.6%, while for wash-water mixes it ranged from 31.5 to 58.9%, indicating substantial early-age strength development in both systems, with comparable or slightly higher gains observed in wash-water mixes. Between 28 and 56 days, the gain varied from –6.1 to 24.0% for potable and 3.7 to 23.9% for wash-water mixes, suggesting that the majority of geopolymerization reactions occurred within the first 28 days, followed by continued secondary gel formation and microstructural refinement. Beyond 56 days up to 90 days, strength gain was minimal (–2.4 to 3.4% for potable and –7.1 to 8.0% for wash water), indicating stabilization of the reaction products and matrix densification. Overall, strength increased sharply up to 28 days and gradually plateaued thereafter, with peak compressive strength generally achieved by 56 days, marking near completion of the principal reaction phases.

**Fig. 10:**
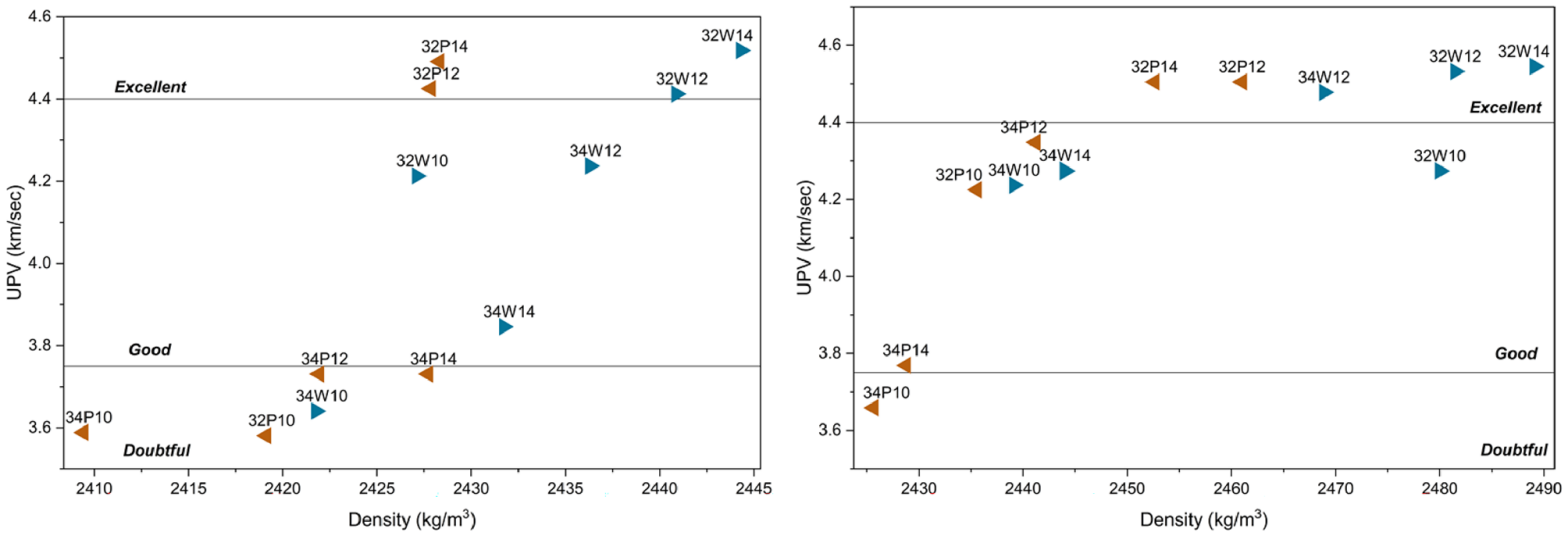
Relationship of Density with Ultrasonic Pulse Velocity at 28 days (left) and 56 days (right) of curing.

Figure 10 illustrates the relationship between density and UPV for all OPAAC mixes at 28 and 56 days of curing. A clear positive correlation is observed as mixes with higher density generally exhibit higher UPV, indicating a denser and more homogeneous internal structure. At 28 days, UPV values range from 3.58 to 4.49 km/s, classifying the mixes from *doubtful* to *excellent* quality. Mixes incorporating wash water, 32W12 and 32W14 display slightly higher UPV than their potable counterparts, suggesting improved matrix consolidation due to the ionic constituents in wash water.

With prolonged curing to 56 days, both density and UPV values increase across all mixes, ranging from 3.66 to 4.55 km/s reflecting continued hydration and pore refinement. The 32W14 and 32W12 mixes record the highest UPV values of 4.54–4.53 km/s, corresponding to *excellent* quality concrete. In contrast, mixes with higher w/b ratios show lower UPV and density, indicating higher porosity and weaker microstructural integrity. Overall, the results confirm that lower w/b ratios, higher activator content, and the use of wash water enhance compactness and internal cohesion, leading to superior UPV performance.

### Split tensile strength (STS) and Flexural strength (FS)

**Fig. 11:**
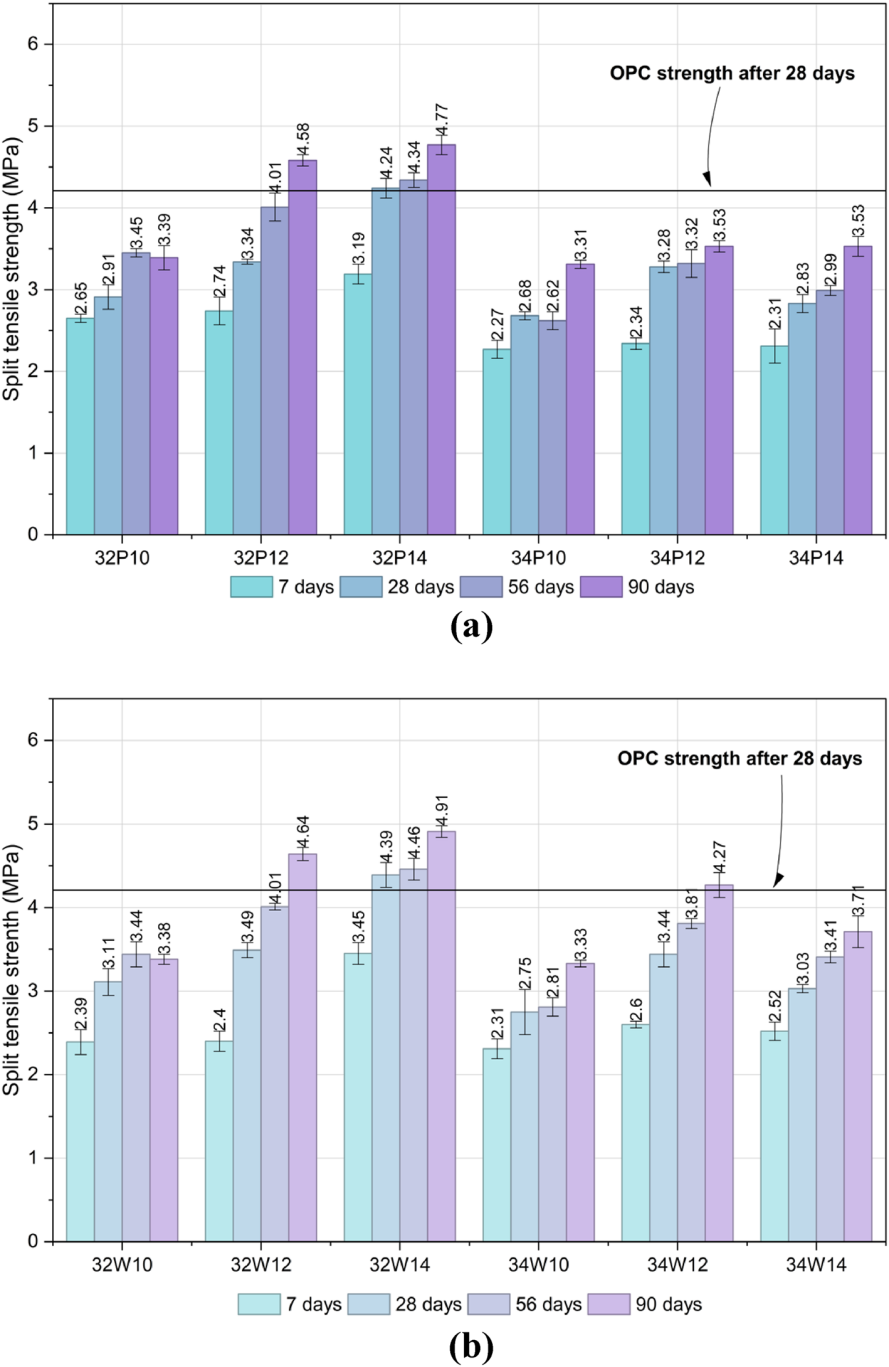
(**a**) Split tensile strength of Potable water mixes at different curing ages. (**b**) Split tensile strength of Wash water mixes at different curing ages.

Figures 11a and b illustrate the influence of activator dosage and w/b ratio on the splitting tensile strength (STS) of OPAAC prepared using potable and wash water, respectively. STS followed trends consistent with compressive strength, increasing with higher activator content and lower w/b ratio. At 28 days, potable-water mixes ranged from 2.7 to 4.2 MPa and wash-water mixes from 2.8 to 4.4 MPa, with 32W14 (4.4 ± 0.15 MPa) marginally exceeding the OPC reference (4.2 MPa).

Two-way ANOVA performed at a 95% confidence level for the strength results at all curing ages. The results confirmed that both w/b ratio and activator dosage significantly influence STS in potable and wash-water systems (*p* < 0.0001). The 28-day results are presented in Tables S8 and S9 (Supplementary Material). The significant interaction effects further indicate that the influence of activator dosage depends on the selected w/b ratio. These results demonstrate that tensile performance is highly sensitive to mix design parameters, with wash water exhibiting comparable or slightly enhanced bond characteristics relative to potable water. Consistent with the compressive strength findings, water type itself did not significantly influence STS, further supporting the suitability of wash water as an alternative mixing medium.

**Fig. 12:**
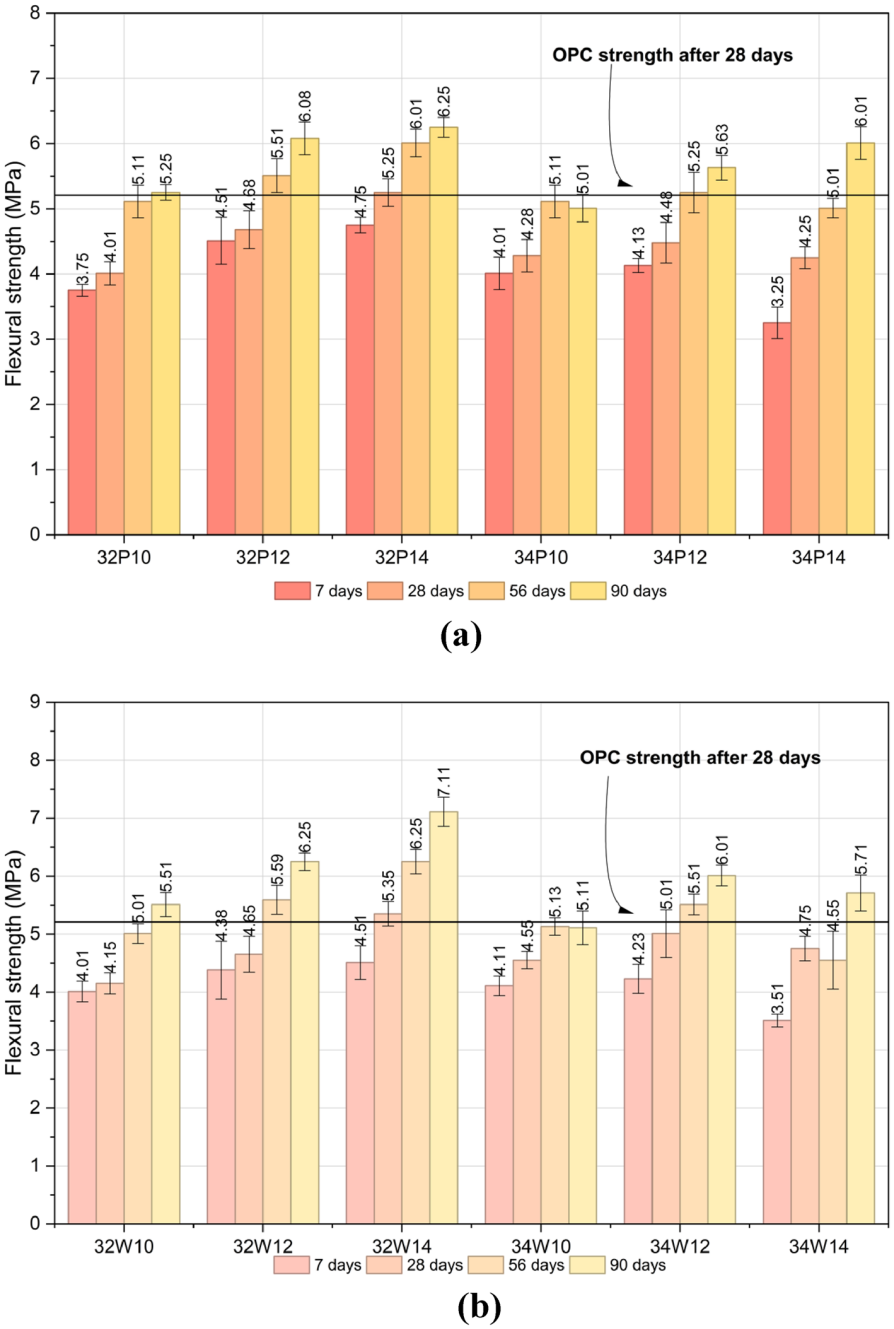
(**a**) Flexural strength of Potable water mixes at different curing ages. (**b**) Flexural strength of Wash water mixes at different curing ages.

Flexural strength is a key parameter for assessing the cracking resistance of concrete under load and deformation in both structural and non-structural applications. It serves as an indirect measure of tensile strength, representing the material’s ability to resist bending failure in unreinforced beams or slabs^44^. Figure 12a and b illustrate the influence of activator content and w/b ratio on the flexural strength of OPAAC prepared with potable and wash water. FS exhibited trends analogous to STS and compressive strength, improving with higher activator content, lower w/b ratio, and longer curing duration. At 28 days, mixes 32P14 and 32W14 achieved flexural strengths of 5.3 ± 0.21 MPa and 5.4 ± 0.21 MPa, respectively, both marginally exceeding the OPC reference of 5.2 MPa.

Two-way ANOVA confirmed that activator dosage significantly influences flexural strength in both potable and wash-water systems (*p* < 0.01). The 28-day results are provided in Tables S10 and S11 (Supplementary Material). The w/b ratio significantly affected FS in potable-water mixes (*p* < 0.05), whereas its influence was not statistically significant in wash-water mixes (*p* > 0.05). The significant interaction effects indicate that the influence of activator dosage depends on the selected w/b ratio. These findings suggest that flexural performance is primarily governed by activator-induced gel formation and matrix densification, while wash water exhibits comparable resistance to potable water.

The STS and FS of OPAAC exhibited trends broadly consistent with compressive strength, as all three mechanical properties are governed primarily by activator dosage, w/b ratio, and curing duration, while mixing water type demonstrated comparable performance across the investigated mixes.

### Comparison between predicted and experimental split tensile and flexural strengths

**Fig. 13:**
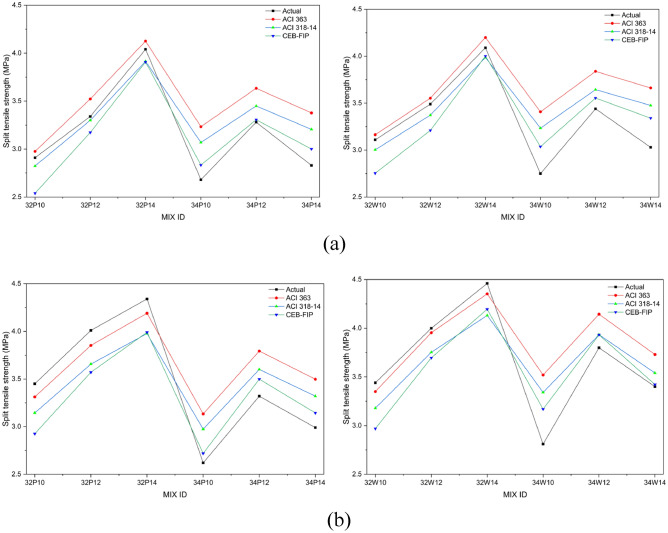
Relationship between Compressive strength and Split Tensile Strength. (**a**) Relationship between CS and STS at 28 days of curing for Potable water (left) and Wash water (right) mixes. (**b**) Relationship between CS and STS at 56 days of curing for Potable water (left) and Wash water (right) mixes.

**Fig. 14:**
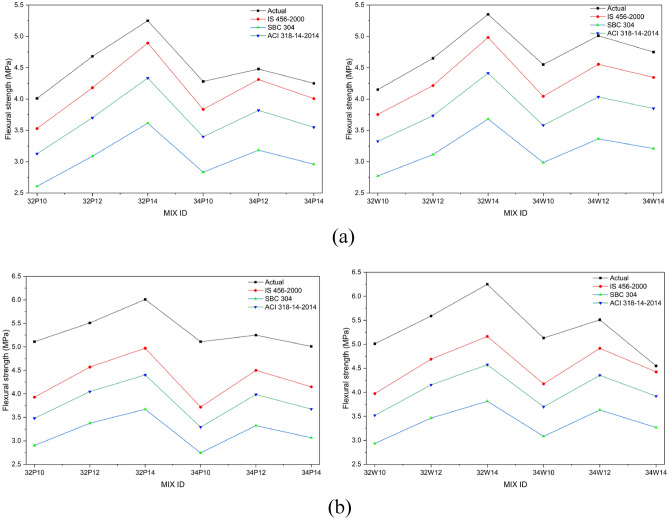
Relationship between Compressive strength and Flexural Strength. (**a**) Relationship between CS and FS at 28 days of curing for Potable water (left) and Wash water (right) mixes. (**b**) Relationship between CS and FS at 56 days of curing for Potable water (left) and Wash water (right) mixes.

Figures 13 and 14 compares the experimental split tensile strength (STS) and flexural strength (FS) with the values predicted by established code-based relationships (IS, SBC, ACI, and CEB-FIP) for mixes with potable water and wash water at 28 and 56 days, respectively. Such comparisons help validate the experimental results against empirical models and assess how closely alkali-activated systems prepared with both potable and wash water follow conventional prediction approaches.

Figure 13a presents the relationship between compressive strength and split tensile strength at 28 days, while Fig. 13b shows the corresponding relationship at 56 days for both potable and wash-water mixes. A quantitative evaluation of the prediction models was performed using the coefficient of determination (R^2^), as summarized in Table 11. Among the evaluated models, the CEB-FIP relationship exhibited the highest correlation with the experimental STS values for both potable- and wash-water mixes at 28 and 56 days (R^2^ = 0.82–0.88). The ACI 363 and ACI 318-14 models also showed good agreement with experimental results, with R^2^ values ranging between 0.80 and 0.87. These results indicate that the CEB-FIP model provides the closest prediction for the split tensile strength of the present alkali-activated concrete mixes. Overall, the relatively high R^2^ values (0.80–0.88) demonstrate that conventional empirical relationships developed for OPC concrete can reasonably predict the tensile performance of the studied alkali-activated systems.

**Table 11:** Coefficient of determination (R^2^) between experimental and predicted STS.

Model	R^2^ (28d PW)	R^2^ (28d WW)	R^2^ (56d PW)	R^2^ (56d WW)
ACI 363	0.81	0.83	0.87	0.86
ACI 318-14	0.8	0.83	0.87	0.85
CEB-FIP	0.82	0.84	0.88	0.88

Figure 14a presents the relationship between compressive strength and flexural strength at 28 days, while Fig. 14b shows the corresponding relationship at 56 days for both potable and wash-water mixes. A quantitative comparison using the coefficient of determination (R^2^) is presented in Table 12. The IS 456-2000 model exhibited the highest correlation with the experimental flexural strength values, with R^2^ values ranging from 0.91 to 0.93 for both potable- and wash-water mixes. The SBC 304 and ACI 318–14 models also showed strong agreement with the experimental data but slightly lower correlations. Overall, the high R^2^ values indicate that conventional empirical relationships developed for OPC concrete can reasonably predict the flexural behavior of the studied alkali-activated concrete mixes.

**Table 12:** Coefficient of determination (R^2^) between experimental and predicted FS.

Model	R^2^ (28d PW)	R^2^ (28d WW)	R^2^ (56d PW)	R^2^ (56d WW)
IS 456-2000	0.91	0.92	0.93	0.93
SBC 304	0.91	0.91	0.92	0.92
ACI 318-14	0.91	0.91	0.93	0.93

### Water absorption

**Fig. 15:**
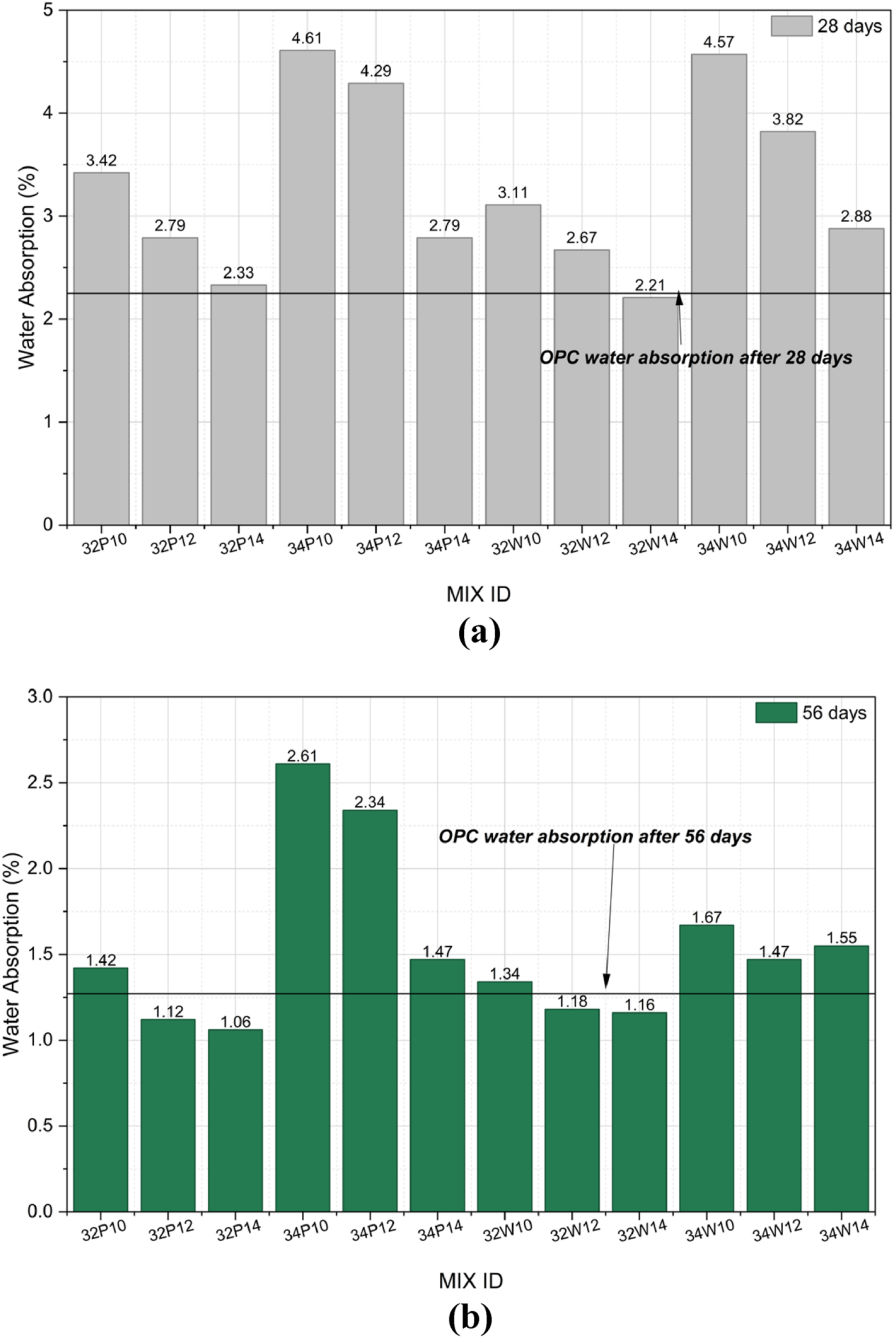
(**a**) Water absorption of OPAAC at 28 days of curing. (**b**) Water absorption of OPAAC at 56 days of curing.

Figure 15a and b shows the water absorption behavior of all mixes at 28 and 56 days of curing. Water absorption values represent the mean of two replicate specimens (n = 2), and variability is expressed as standard deviation, as shown by the error bars in respective figures. At 28 days, absorption ranged from 2.21 to 4.61%, reducing to 1.06–2.61% at 56 days, indicating progressive pore refinement and matrix densification with age. Mixes with a lower w/b ratio of 0.32 consistently exhibited lower absorption than those with a higher ratio of 0.34, owing to reduced water content that limits capillary voids and promotes a finer, discontinuous pore structure, thereby decreasing permeability and enhancing matrix compactness^45^. Furthermore, water absorption decreased with increasing activator content for both mixing waters, attributed to improved dissolution of the aluminosilicate precursors and enhanced formation of C/N–A–S–H gels, resulting in a denser microstructure with reduced porosity, consistent with previous findings^46^.

The highest water absorption was recorded for mix 34P10, with values of 4.61% and 2.61% at 28 and 56 days, respectively, attributed to its higher w/b ratio and lower activator content, which likely led to incomplete hydration and a more porous matrix. In contrast, the lowest absorption values were observed for 32P14 with 2.33% among potable water mixes and 32W14 with 2.21% among wash water mixes at 28 days, with the latter performing slightly better than the OPC control of 2.25%. At 56 days, these mixes exhibited further reductions to 1.06% and 1.16%, respectively, both below the OPC control value of 1.27%, indicating impermeability and a well-developed gel network. Overall, all mixes showed a pronounced decline in water absorption with curing age, particularly those prepared with wash water, suggesting that its ionic constituents promote enhanced matrix densification and reduced permeability.

### Rate of absorption (sorptivity) of water

**Fig. 16:**
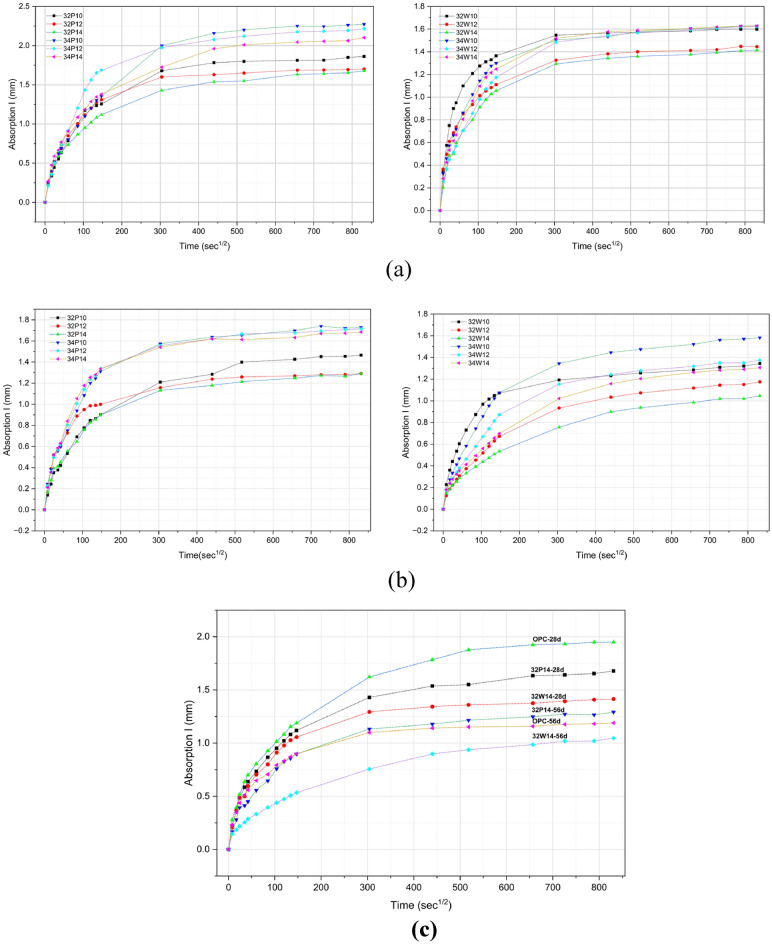
Sorptivity results of Potable water and Wash water mixes. (**a**) Sorptivity of Potable water (left) and Wash water (right) mixes at 28 days of curing. (**b**) Sorptivity of Potable water (left) and Wash water (right) at 56 days of curing. (**c**) Sorptivity of mixes with 14% activator (0.32 w/b) and OPC at 28 and 56 days of curing.

Sorptivity quantifies the rate of water absorption through capillary pores, an indicator of pore structure and tortuosity. In OPAAC, lower sorptivity reflects a denser matrix with more tortuous capillary pathways, thereby improving resistance to water ingress and enhancing durability^47^. The sorptivity values of all mixes at 28 and 56 days of curing are presented in Fig. 16a and b, respectively.

Mixes prepared with wash water exhibited slightly lower sorptivity across all activator levels, attributed to matrix densification. Higher w/b ratios resulted in greater sorptivity due to increased inter-particle voids for both water types. A consistent reduction in absorption from 28 to 56 days indicated progressive pore refinement and improved durability, aligning with previous findings^48^. All mixes showed an initial rapid absorption phase, followed by stabilization, reflecting early-stage capillary suction, while gel pores gradually reduce the absorption rate at later stages^49^. Higher activator content of 14% yielded lower sorptivity than 10% and 12% mixes, owing to enhanced binder reaction and gel formation. The combination of a lower w/b ratio and higher activator content produced the densest microstructure, as evident in the reduced sorptivity of 32P14 and 32W14 at both curing ages.

Figure 16c presents only mixes 32P14, 32W14, and OPC to compare sorptivity behaviour of potable water, wash water, and control concrete, while the complete sorptivity coefficients for all mixes are provided in Tables S12 and S13, with the corresponding values summarized in Table 13. The slopes obtained from the linear regression of cumulative absorption (I) versus the square root of time (√t) correspond to the initial sorptivity (Si) and secondary sorptivity (Ss) coefficients. Among the investigated mixes, 32W14-28d exhibited the lowest secondary sorptivity, indicating reduced long-term capillary transport compared with the OPC control. At 56 days, both Si and Ss generally decreased due to continued reaction and progressive pore refinement, with 32W14-56d showing the lowest initial sorptivity (4.93 × 10^−3^ mm/s^1/2^). The reduction in secondary sorptivity at later ages reflects decreased pore connectivity and slower water ingress through the hardened matrix. Wash-water mixes exhibited sorptivity values comparable to those of potable-water mixes, with slightly lower secondary sorptivity indicating marginally improved resistance to long-term water ingress. Overall, OPAAC mixes demonstrated superior sorptivity performance compared to the OPC control, as illustrated in Fig. 16 (c). The water absorption ranking at both curing ages is as follows: 32W14-56 < OPC-56 < 32P14-56 < 32W14-28 < 32P14-28 < OPC-28.

**Table 13:** Sorptivity coefficients (initial and secondary) concrete mixes.

Mix ID	Initial S_i_ (mm/s^1/2^)	Secondary S_s_ (mm/s^1/2^)
OPC-28d	6.90 × 10^–3^	5.76 × 10^–4^
32P14-28d	6.60 × 10^–3^	2.72 × 10^–4^
32W14-28d	6.27 × 10^–3^	2.13 × 10^–4^
32P14-56d	5.39 × 10^–3^	1.94 × 10^–4^
OPC-56d	5.03 × 10^–3^	1.51 × 10^–4^
32W14-56d	4.93 × 10^–3^	1.36 × 10^–4^

## Microstructure

### XRD analysis

**Fig. 17:**
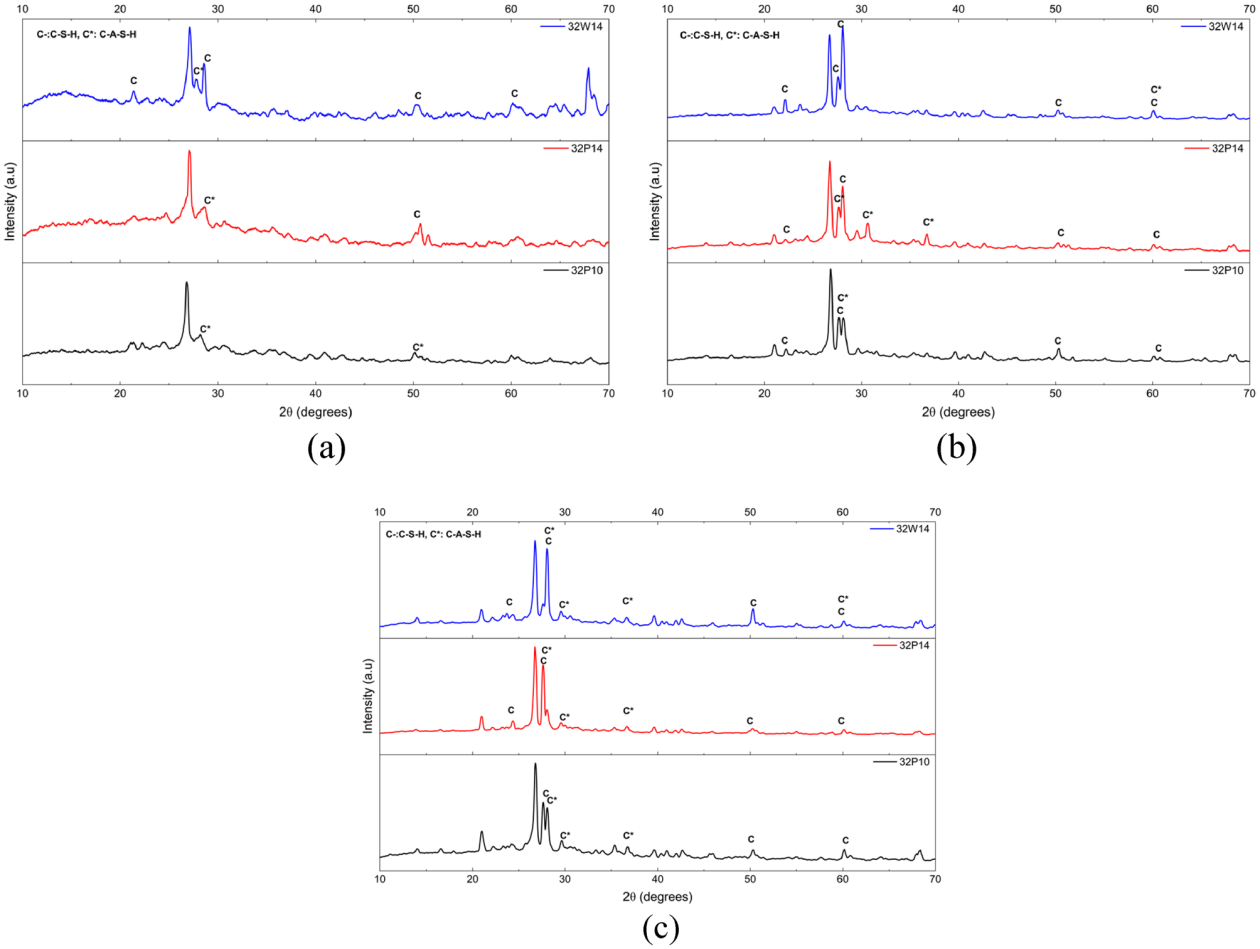
XRD patterns of OPAAC after curing for different ages: (**a**) 7 days; (**b**) 28 days and (**c**) 56 days.

To investigate the reaction mechanism of OPAAC, three representative mixes, namely 32P10, 32P14, and 32W14, were examined by XRD after 7, 28, and 56 days of ambient curing, as shown in Fig. 17a–c. The crystalline phases identified include quartz, with a characteristic reflection at 2θ ≈ 26.6° (ICDD PDF 96-101-1171), and calcite, at 2θ ≈ 29.4° (ICDD PDF 96-900-0968). Quartz represents a residual crystalline phase originating mainly from the unreacted fly ash particles, whereas calcite is attributed to the carbonation of Ca-bearing phases present in the alkali-activated matrix.

The principal binding phases in alkali-activated systems, namely C–A–S–H and N–A–S–H gels, are largely amorphous or poorly crystalline, and therefore appear as a broad diffuse halo in the 20°–35° (2θ) region rather than as distinct diffraction peaks. XRD phase matching performed using HighScore Plus further indicated the presence of a tobermorite-like calcium silicate hydrate phase (ICDD PDF 96-900-2246) at 2θ ≈ 28.9°, 32.0°, and 49.9°, suggesting the formation of C–S–H/C–A–S–H type reaction products associated with the calcium supplied by the slag component. A zeolitic sodium aluminosilicate phase (Na_6_(Al_6_Si_10_O_32_)(H_2_O)_12_; ICDD PDF 96-900-5061) is indicated by weak reflections near 2θ ≈ 21.7° and 28.1°, consistent with secondary crystallisation of aluminosilicate gel during hydration as the network gradually reorganises into more ordered frameworks.

At 7 days of curing, the XRD patterns exhibit a relatively broad and low-intensity diffuse halo between 20° and 35° (2θ), indicating the early formation of poorly polymerized aluminosilicate and calcium aluminosilicate gel phases. Among the mixes, 32P10 shows the least pronounced halo, suggesting comparatively lower reaction extent at the lower activator dosage, whereas 32P14 exhibits a slightly more developed hump due to the higher activator content. With increasing curing age to 28 days, the diffuse hump becomes more pronounced, suggesting continued hydration and increased formation of amorphous reaction products, accompanied by partial crystallization of calcium-rich phases as observed in 32P14 and 32W14. By 56 days, the persistence of the amorphous halo together with slightly intensified calcite reflections indicates progressive development and densification of the binder matrix. The mixes 32P14 and 32W14 exhibit a more pronounced diffuse hump, suggesting a more developed reaction product structure compared with 32P10. Notably, certain minor crystalline peaks observed at 3 days diminished by 28 days, suggesting the progressive dissolution of precursor phases and the formation of amorphous gel products as the alkali-activation reaction progressed^50^. It should be noted that quantitative phase analysis through Rietveld refinement was not performed in this study; the XRD observations presented are therefore qualitative in nature.

### SEM–EDS analysis

**Fig. 18:**
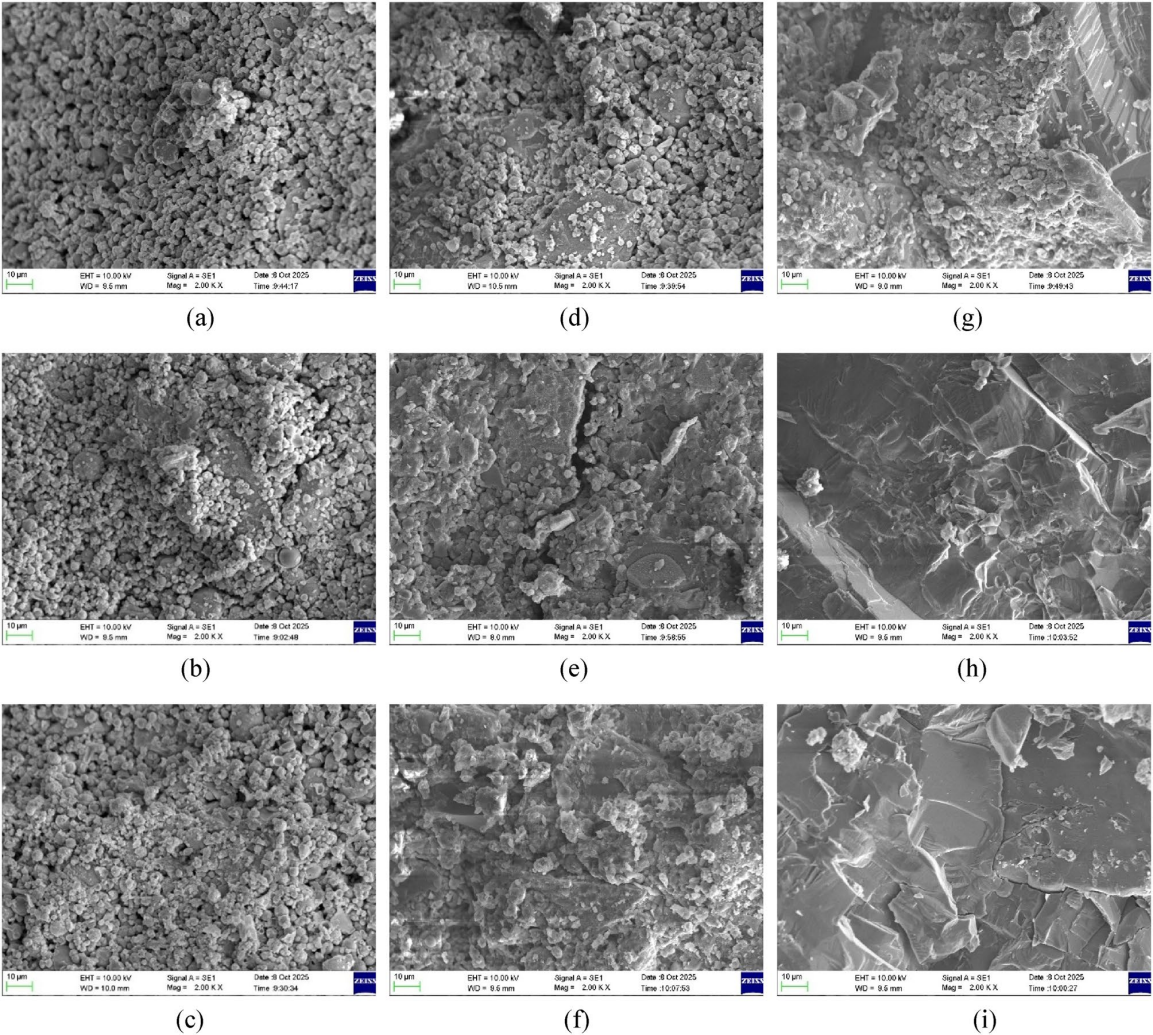
SEM images of OPAAC at curing different ages (2000 × magnification). (**a**) 32P10- 7 days, (**b**) 32P14- 7 days, (**c**) 32W14- 7 days, (**d**) 32P10- 28 days, (**e**) 32P14- 28 days, (**f**) 32W14- 28 days, (**g**) 32P10- 56 days, (**h**) 32P14- 56 days, (**i**) 32W14- 56 days

Figure 18 shows the SEM micrographs of selected mixes 32P10, 32P14, and 32W14 after 7, 28, and 56 days of curing, illustrating the influence of activator content and water type on microstructural development. Figure 18a, b, and c shows the microstructure at 7 days for 32P10, 32P14, and 32W14, respectively. All mixes show unreacted FA spheres and angular GGBS particles, indicating the early stage of geopolymerization along with the presence of (N, C)–A–S–H gel and flaky crystalline phases.

Figure 18d, e, and f shows the microstructures at 28 days. Compared with the corresponding 7-day, the formation of (N, C)–A–S–H gel and flaky crystals become more pronounced, with fewer voids and a denser matrix. This densification reflects the progressive dissolution of FA and GGBS and the formation of reaction products that contribute to strength development. Previous studies using the same precursors^51^ and activators^52^ reported that the coexistence of C–A–S–H and N–A–S–H phases govern the 28-day strength, which aligns with the current microstructural observations.

With curing age, the microstructures of all three OPAA mixtures become dense by the reduction of the number of micro-pores as seen in Fig. 18h and i at 56 days of curing. This is due to the formation of new hydration products, mainly calcium-sodium aluminosilicate hydrate (C–(N)–A–S–H)-type gel, which was confirmed using EDS point analysis on the reacted phase in the hardened binder^53^. Figure 18h and i show a markedly denser and more homogeneous microstructure than Fig. 18g, due to higher activator content which aligns with their higher compressive strength.

Quantitative EDS analysis was performed on the selected mixes at 7, 28, and 56 days to evaluate the reaction products and elemental composition of the hardened binders. The atomic ratios presented in Table 14 represent the average of five measurement spots (reported as mean ± standard deviation), and confirm the presence of calcium (Ca), silicon (Si), aluminum (Al), sodium (Na), and oxygen (O) across all samples. In the FA–GGBS-based one-part system, elevated concentrations of Ca, Si, and Na facilitate the formation of flaky crystalline products and N–(C)–A–S–H-type gels^51,54^.

**Table 14:** Elements of selected mixes in atomic %

Curing age	7 Days		
Element	32P10	32P14	32W14
Na	3.05 ± 0.94	3.16 ± 1.05	3.12 ± 1.12
Al	9.14 ± 2.15	7.65 ± 0.73	7.51 ± 2.08
Si	20.36 ± 3.26	21.64 ± 2.61	22.48 ± 3.75
Ca	2.68 ± 0.78	2.81 ± 0.71	2.98 ± 1.35
Si/Al	2.23	2.83	2.99
Ca/Si	0.13	0.13	0.13
Na/Al	0.33	0.41	0.42

	28 Days		
	32P10	32P14	32W14
Na	3.24 ± 0.54	3.94 ± 0.32	3.93 ± 0.46
Al	7.97 ± 1.16	7.01 ± 0.53	7.03 ± 1.52
Si	22.90 ± 1.91	27.11 ± 0.35	28.10 ± 0.78
Ca	3.34 ± 0.75	5.13 ± 0.62	5.57 ± 0.62
Si/Al	2.87	3.87	4
Ca/Si	0.15	0.19	0.2
Na/Al	0.41	0.56	0.56

	56 Days		
	32P10	32P14	32W14
a	3.46 ± 1.03	4.52 ± 0.38	4.63 ± 1.11
Al	8.12 ± 2.01	7.00 ± 0.17	6.95 ± 2.07
Si	23.13 ± 3.40	27.72 ± 0.75	27.99 ± 2.93
Ca	4.62 ± 1.15	5.83 ± 1.18	6.21 ± 1.74
Si/Al	2.85	3.96	4.03
Ca/Si	0.2	0.21	0.22
Na/Al	0.43	0.65	0.67

A gradual increase in Si and Ca contents from 7 to 56 days can be observed for most mixes. This trend indicates ongoing reaction and progressive development of the binder structure with curing time. The continued increase in Ca content, particularly in the higher-activator mixes, suggests enhanced formation of calcium-containing reaction products. Lower Si/Al ratios, as observed in 32P10 at early ages, indicate a relatively less polymerised aluminosilicate network. In contrast, the higher ratios observed in 32P14 and 32W14 at later ages reflect greater incorporation of silicate species into the aluminosilicate framework, leading to a more polymerised and cross-linked gel network. This enhanced polymerisation contributes to improved microstructural densification and mechanical performance^55^. The Ca/Si ratio increases gradually with curing age, reflecting the growing contribution of calcium to the reaction products over time, likely due to the progressive participation of slag in the reaction process. Higher Ca/Si ratios in mixes with greater activator dosage indicate a higher degree of calcium involvement in the gel phase. The concurrent increase in both Ca/Si and Si/Al ratios suggests the coexistence of C–(A)–S–H and N–A–S–H gels, which contributes to enhanced matrix densification. These values fall within the range typically associated with hybrid C–(N)–A–S–H type gels in FA–GGBS systems, suggesting partial integration of calcium into the aluminosilicate network^56^. The Na/Al ratio indicates that sodium acts primarily as a charge-balancing cation for the aluminosilicate framework. The moderate increase in Na/Al ratio for mixes with higher activator dosage suggests greater availability of alkali ions during the activation process. The values remain well below 1.0 across all mixes and ages, confirming that Ca^2+^ co-participates in charge-balancing alongside Na^+^, consistent with the hybrid gel character of the system.

A comparison between mixes 32P14 and 32W14 indicates minor differences in elemental composition. The 32W14 mix exhibits slightly higher Si and Ca contents as well as marginally higher Si/Al and Ca/Si ratios at later curing ages. These differences suggest a somewhat greater incorporation of silicate and calcium species into the reaction products, indicating a slightly more polymerised gel structure. However, the overall compositional trends between the two mixes remain similar, which is consistent with the statistical analysis showing that the effect of water type on mechanical performance was not significant. These compositional trends are also consistent with the SEM observations, where progressive microstructural densification and reduced unreacted particles were evident with increasing age.

## Equivalent CO_2_ emission and cost evaluation

The assessment follows a cradle-to-gate approach, encompassing raw material extraction, processing, manufacturing, and transportation of each constituent from its source to the mixing place. The carbon emission factors for the constituent materials and their transportation distances used in the calculations are presented in Table 15. The wash water used in this study did not require any additional chemical treatment prior to mixing; therefore, no additional CO_2_ emissions were considered for its use^57^. The emission factors adopted represent the embodied CO_2_ associated with the complete production of the materials and were obtained from peer-reviewed life-cycle assessment (LCA) databases^58^. These factors include the energy-intensive production of alkaline activators such as sodium silicate and sodium hydroxide, ensuring that their manufacturing emissions are fully reflected in the carbon footprint estimation. The data indicate that Portland cement and alkali activators exhibit considerably higher CO_2_ emissions per unit mass compared to aluminosilicate precursors. However, the quantity of activators required to produce 1 m^3^ of OPAA concrete is substantially lower than the amount of cement used in OPC concrete.

**Table 15:** Carbon footprint of materials used in and concrete production.

Material	Carbon emission (tonne CO_2_-e/tonne)	Road distance to destination (km)
Fly Ash	0.027	413
Slag (GGBS)	0.143	65
OPC Cement	0.86	2
Sodium Silicate	0.892	750
Sodium Hydroxide	0.625	750
Coarse Aggregate	0.036	5
Fine Aggregate	0.014	5
Wash water	–	5

The total CO_2_ emissions associated with producing each concrete mix were calculated using Eq. (1), considering 1 m^3^ of concrete for all mixes:

1 $${\mathrm{E}}_{{{\mathrm{e}} - {\mathrm{CO2}}}} = \sum \left( {(w_{i} /1000){ } \times CEF} \right) + E_{{Fmix - CO_{2} }}$$

where $E_{{e - CO_{2} }}$ is the CO_2_ emission associated with material production, expressed in kg CO_2_/m^3^ of concrete; $w_{i}$ is the mass of each material in kg/m^3^; $CEF_{i}$ is the carbon emission factor of each material in kg CO_2_/tonne; and $E_{{Fmix - CO_{2} }}$ is the emission associated with concrete mixing, taken as 1.61 kg CO_2_/m^3^^59^.

The transport-related CO_2_ emissions were calculated using Eq. (2):

2 $${\mathrm{E}}_{{{\mathrm{t}} - {\mathrm{CO2}}}} = \sum \left( {(w_{i} /1000){ } \times di} \right) \times E_{{Fuel - CO_{2} }}$$

where $E_{{t - CO_{2} }}$ is the CO_2_ emission associated with transportation, expressed in kg CO_2_/m^3^ of concrete; $w_{i}$ is the mass of each material in kg/m^3^; $d_{i}$ is the transport distance in km; and $E_{{Fuel - CO_{2} }}$ is the emission factor for loaded diesel heavy goods trucks, taken as 0.062 kg CO_2_/(tonne km)^60^.

The calculated embodied CO_2_ emissions for each material component in the concrete mixes are summarized in Table 16. For the OPAAC mix, the total embodied CO_2_ emission was 139.36 kg/m^3^, when potable water was considered (32P14), the total CO_2_ emissions decreased slightly to 139.20 kg CO_2_/m^3^. The contribution of wash water to the overall emissions was negligible because it is a recycled by-product generated within the RMC plant. In contrast, the OPC concrete exhibited a significantly higher total embodied CO_2_ emission of 427.34 kg/m^3^, primarily due to the high carbon intensity associated with cement production. Cement alone accounted for approximately 373.29 kg/m^3^, representing the dominant contributor to the overall emissions of the OPC system. Overall, the OPAAC system demonstrated a substantial reduction in CO_2_ emissions of approximately 67% compared with conventional OPC concrete of similar strength grade.

**Table 16:** Total embodied CO_2_ emissions of OPAAC and OPC (kg/m^3^).

Material	Quantity (kg/m^3^)	Transport CO_2_ (kg/m^3^)	Production CO_2_ (kg/m^3^)	Total CO_2_ (kg/m^3^)
OPACC CO_2_ emissions				
Fly Ash	337.5	8.64	9.11	17.75
Slag (GGBS)	112.5	0.45	16.0875	16.5375
Sodium Silicate	42	1.95	37.464	39.414
Sodium Hydroxide	21	0.98	13.125	14.105
Coarse Aggregate	1129	0.35	40.644	40.994
Fine Aggregate	614	0.19	8.596	8.786
Wash water	144	0.16	–	0.16
Mixer	–	–	1.61	1.61
Total				139.3565
32W14			139.3565	
32P14		139.3565–0.16	139.1965	
OPC CO_2_ emissions				
OPC	21	0.05	373.24	373.29
Coarse Aggregate	1196	0.35	43.056	43.406
Fine Aggregate	632	0.19	8.848	9.038
Mixer	–	–	1.61	1.61
Total				427.344

**Fig. 19:**
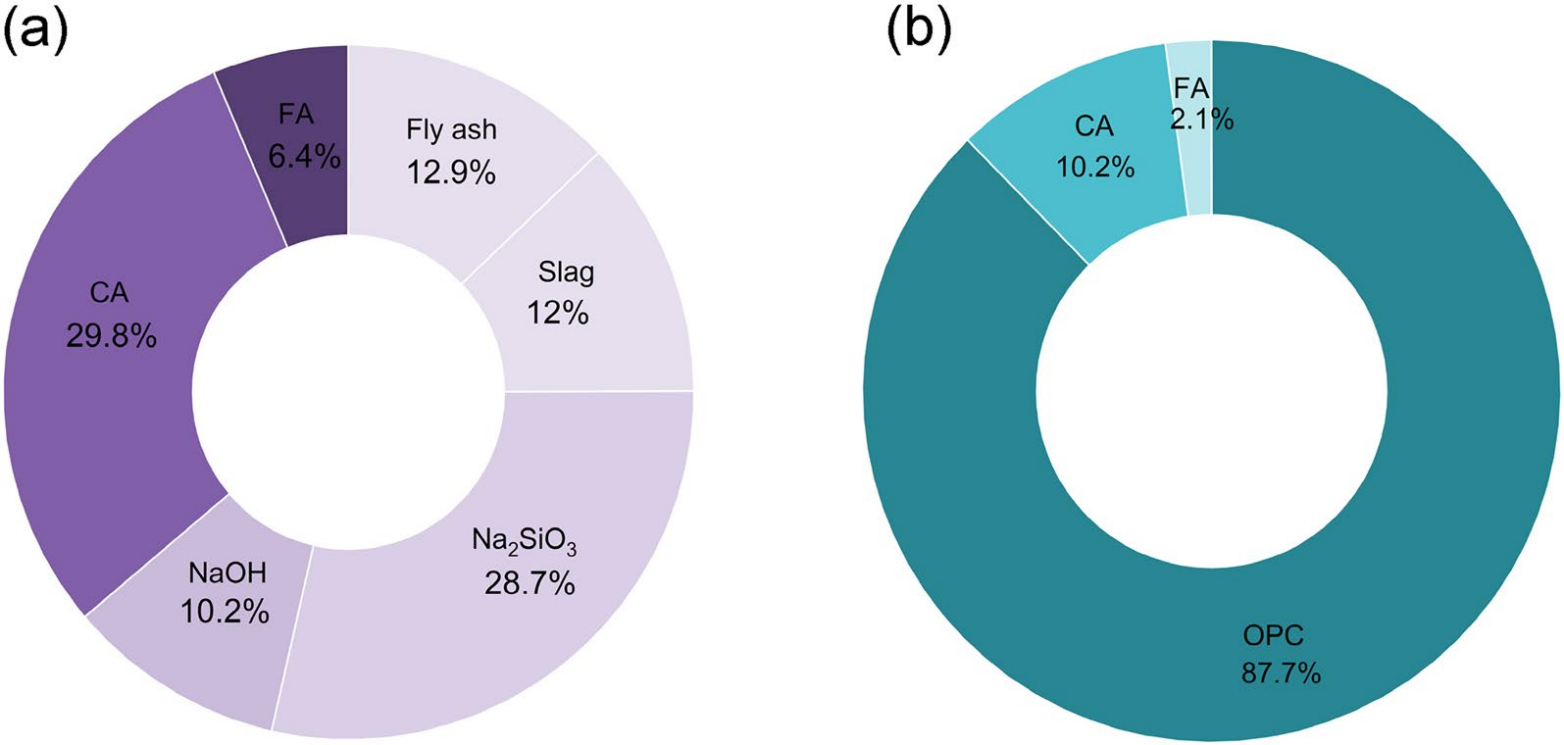
Contributions of materials to carbon footprints of (**a**) OPAAC and (**b**) OPCC.

Figure 19 illustrates the percentage contribution of different materials to the total embodied CO_2_ emissions of (a) OPAAC and (b) OPC concrete. In OPAAC, the primary contributors are the alkaline activators, along with aggregates, whereas in OPC concrete nearly 87% of the total emissions originate from Portland cement production. This indicates that although the use of industrial by-products such as fly ash and slag significantly reduces clinker-related emissions, the carbon footprint in alkali-activated systems is mainly associated with the production of alkaline activators and aggregates. Overall, these results highlight the substantial potential for emission reduction through the utilization of aluminosilicate-based binders, reinforcing the role of one-part alkali-activated systems in advancing sustainable concrete production.

A sustainable concrete mix must also be economically viable to facilitate its in-situ application and commercial adoption. Accordingly, a comparative cost analysis was carried out for the OPAA and OPC concrete mixes investigated in the present study, considering only the material costs and transportation costs. The results of the cost evaluation are presented in Table 17. Material costs are based on prevailing market prices in southern India (INR) for the financial year 2023–2024, obtained from local suppliers. It is acknowledged that activator costs may vary significantly by region and procurement volume. For all other materials, transport costs were estimated at INR 2.5/tonne·km, a standard bulk road freight rate in Karnataka^61^.

**Table 17:** Total cost summary of OPAAC and OPCC (INR/m^3^).

Material	Quantity (kg/m^3^)	Rate (INR/kg)	Material cost (INR/m^3^)	Distance (km)	Transportation cost (INR/m^3^)
OPAAC cost					
Fly Ash	337.5	1	337.5	413	348.47
Slag (GGBS)	112.5	5	562.5	65	18.28
Sodium Silicate	42	35	1470.00	750	78.75
Sodium Hydroxide	21	85	1785.00	750	39.38
Coarse Aggregate	1129	2.17	2449.93	5	14.11
Fine Aggregate	614	1.83	1123.62	5	7.67
Wash Water	144			5	144*
Total			*7728.55*		*650.66*
32W14					8379.21
32P14			8379.21–144		8235.21
OPCC cost					
OPC Cement	434	6.6	2864.40	2	2.17
Coarse Aggregate	1196	2.17	2595.32	5	14.95
Fine Aggregate	632	1.83	1156.56	5	7.9
Total			*6616.28*		*25.02*
OPCC					6641.3

*Fixed INR 1000/trip.

**Fig. 20:**
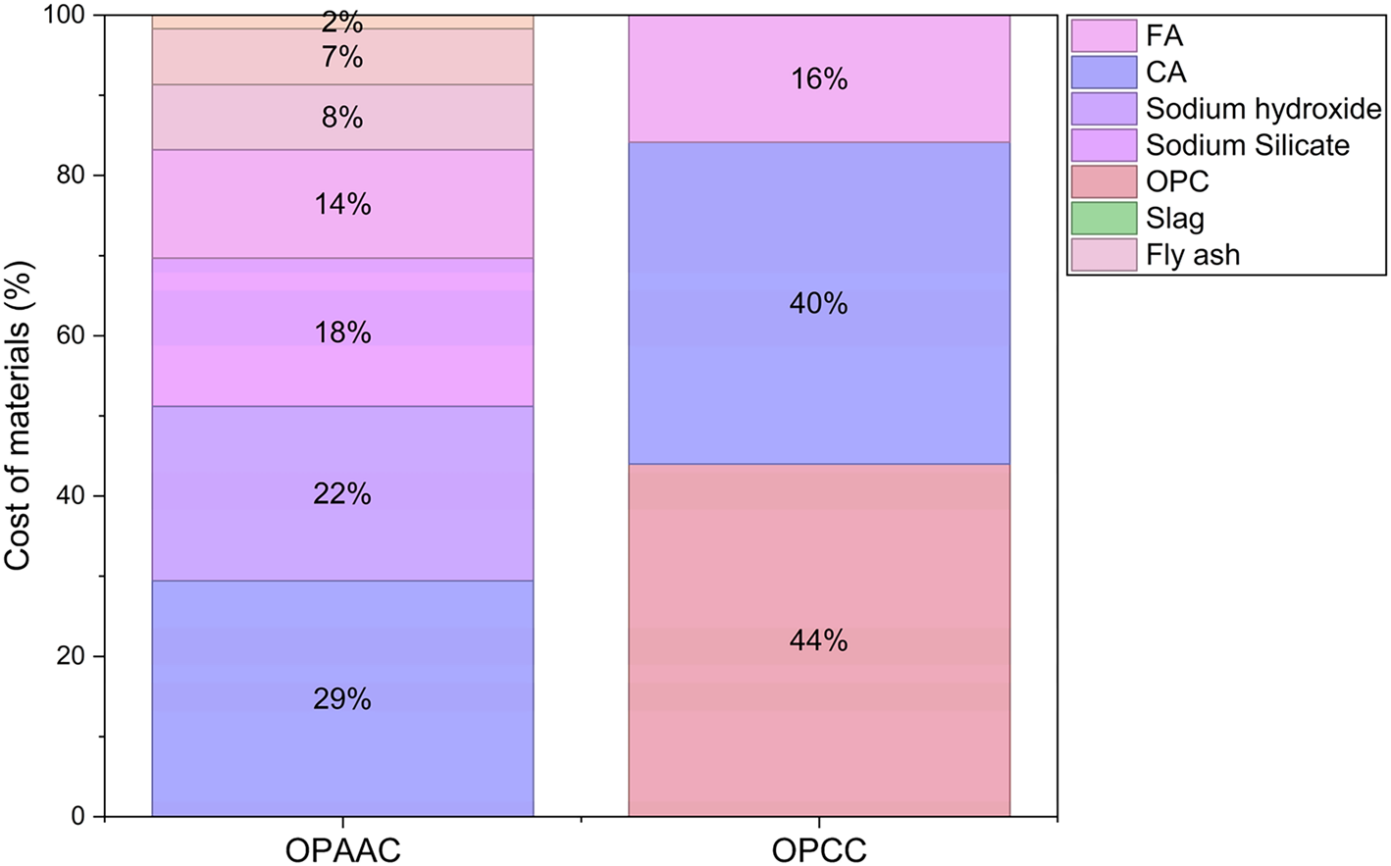
Cost contribution of materials in OPAA and OPC concrete (%).

The total cost of OPAAC (32W14) is INR 8379.21/m^3^, which decreases slightly to INR 8235.21/m^3^ when potable water is used (32P14). In contrast, the total cost of OPCC is INR 6641.30/m^3^, with cement being the dominant cost component, followed by aggregates. Overall, OPAAC is approximately 26% more expensive than OPCC, mainly due to the higher cost and transportation of alkaline activators. Figure 20 shows that the cost of OPAA concrete is primarily governed by sodium hydroxide and sodium silicate (~ 40%), whereas cement dominates the cost of OPC concrete (~ 44%). These results indicate that the cost drivers differ between the two systems, with activators governing the cost of OPAA concrete and cement dominating the cost of OPC concrete.

Previous studies have reported that alkali-activated materials are typically 7–39% more expensive than conventional OPC concrete, primarily due to the cost of alkaline activators^62^. Geopolymer concrete has been shown to reduce greenhouse gas emissions by approximately 12–50% depending on the strength class, although its production cost may be higher than Portland cement concrete because of activator manufacturing^63^. Similarly, alkali-activated concrete produced using commercial activators can achieve about 64% lower global warming potential than OPC, which may increase to around 70% when waste-glass-derived silicate activators are used^64^. Fly ash–GGBFS-based alkali-activated concrete has also been reported to exhibit about 35% lower CO_2_ emissions compared with OPC Life cycle assessment of alkali-activated concretes under marine exposure in an Australian context^65^. However, alkaline activators may contribute up to 74% of the total greenhouse gas emissions in geopolymer systems, indicating that the environmental advantage depends strongly on the type and quantity of activator used^66^.

The results of the present study are consistent with these findings, as the developed OPAAC mix showed approximately 67% lower CO_2_ emissions than OPC concrete of similar strength grade, although the production cost was about 26% higher, mainly due to the use of alkaline activators. Nevertheless, when evaluated in terms of cost per unit reduction in CO_2_ emissions, OPAA concrete demonstrates favourable environmental performance and represents a promising sustainable alternative to conventional OPC concrete.

## Conclusion

1. The incorporation of RMC wash water improved the workability of one-part alkali-activated (OPAA) concrete by 14–29% compared with potable-water mixes, which can be attributed to the higher ionic concentration of wash water that enhances particle dispersion and reduces interparticle friction.
2. Mechanical strength parameters- compressive, tensile, and flexural strength increased with decreasing w/b ratio and increasing activator content, with wash-water mixes outperforming potable-water mixes at all curing ages.
3. The 32P14 mix prepared with potable water achieved a 28-day compressive strength of 48.89 MPa, satisfying the target strength requirement for M40-grade concrete, while the corresponding wash-water mix (32W14) further improved performance by attaining 50.67 MPa at 28 days, thereby demonstrating the feasibility and performance advantage of using wash water in OPAA concrete.
4. Durability indicators such as UPV, density, water absorption, and sorptivity confirmed that a 14% activator dosage combined with a w/b ratio of 0.32 produced a denser, less permeable matrix, with wash-water mixes consistently outperforming potable-water mixes. Microstructural analysis corroborated these findings, showing denser gel formation and higher Ca/Si and Si/Al ratios in wash-water mixes, indicative of enhanced polymerization and matrix refinement.
5. From an environmental and economic perspective, OPAA concrete achieved approximately 67% lower CO_2_ emissions than OPC concrete, despite a moderate 26% increase in material cost. The substitution of potable water with RMC wash water contributed negligibly to the total CO_2_ emissions and therefore did not affect the overall environmental advantage.

## Limitations

- Durability-related tests such as water absorption and sorptivity were conducted using two specimens (n = 2). Although the observed variability was within acceptable limits, the smaller sample size for durability testing may limit the statistical robustness of those results. Future investigations may incorporate a larger number of specimens to further strengthen statistical reliability.
- In this study, the ASTM C642 test was used to determine water absorption after immersion; however, permeable porosity and void volume were not evaluated and could be considered in future investigations for a more comprehensive characterization of the pore structure.
- The present study utilised a single batch of wash water collected from one RMC plant to ensure experimental consistency. However, the composition of wash water may vary across different plants and production cycles; therefore, further studies considering multiple sources and batches of wash water are recommended.

## Future scope

- Long-term durability studies should be conducted under aggressive environments such as chloride, sulfate, and acidic exposures to confirm the performance of OPAAC incorporating wash water under real-world service conditions.
- While this study considered only raw material-related CO_2_ emissions and material costs, a detailed life-cycle assessment, including transportation, energy use, and wash water utilization, is recommended to fully quantify the environmental and resource benefits of OPAA concrete.
- Further research can explore the use of other non-potable water sources such as treated greywater or effluents in alkali-activated systems to broaden sustainable water alternatives for concrete production.

## Supplementary Information

Supplementary Information.

## Data Availability

The datasets generated during and/or analysed during the current study are available from the corresponding author on reasonable request.
