# Supplementary material for: Mechanical performance of one part alkali activated concrete incorporating RMC wash water
Source: Sci Rep. 2026 Apr 6;16:16615. doi: 10.1038/s41598-026-46730-4 (PMC13219767; doi:10.1038/s41598-026-46730-4)
Supplement: Supplementary file 1 — Supplementary Information. [file 41598_2026_46730_MOESM1_ESM.docx]

**SUPPLEMENTARY MATERIAL**

**Table S1 Preliminary mix compositions and results used for selection of final OPAAC proportions**

| Trial No. | FA: GGBS | w/b | Activator (SS:SH-2:1) | Slump mm | Compressive strength Mpa (28 days) |
| --- | --- | --- | --- | --- | --- |
| 1 | 80/20 | 0.28 | 12% | < 25 | 29.3 |
| 2 | 75/25 | 0.28 | 12% |  | 36.0 |
| 3 | 70/30 | 0.28 | 12% |  | 43.0 |
| 4 | 65/35 | 0.28 | 12% |  | 55.2 |
| 5 | 75/25 | 0.35 | 12% | >150 | 34.0 |
| 6 | 75/25 | 0.30 | 8% | 63 | 22.4 |
| 7 | 75/25 | 0.30 | 12% | 50 | 40.3 |
| 8 | 75/25 | 0.30 | 16% | 32 | 60.4 |

**Table S2 Compressive Strength Data**

| **Mix** | **7 days** | **28 days** | **56 days** | **90 days** |
| --- | --- | --- | --- | --- |
| 32P10 | 19.8 ± 0.61 | 25.4 ± 0.40 | 31.5 ± 0.99 | 30.8 ± 0.92 |
| 32P12 | 26.3 ± 0.83 | 35.75 ± 0.15 | 42.6 ± 0.52 | 44.1 ± 1.01 |
| 32P14 | 35.8 ± 0.21 | 48.9 ± 0.36 | 50.4 ± 1.13 | 50.6 ± 0.11 |
| 34P10 | 18.9 ± 0.38 | 30.0 ± 0.70 | 28.2 ± 1.21 | 32.4 ± 0.24 |
| 34P12 | 26.6 ± 0.31 | 37.9 ± 0.36 | 41.3 ± 1.26 | 42.4 ± 0.04 |
| 34P14 | 23.1 ± 0.91 | 32.8 ± 0.20 | 35.2 ± 1.04 | 36.3 ± 1.22 |
| 32W10 | 21.9 ± 0.21 | 28.8 ± 0.95 | 32.2 ± 1.06 | 31.1 ± 1.32 |
| 32W12 | 26.4 ± 0.41 | 36.3 ± 0.15 | 44.9 ± 1.21 | 45.4 ± 1.09 |
| 32W14 | 39.7 ± 0.81 | 50.7 ± 0.44 | 54.4 ± 1.07 | 55.1 ± 1.05 |
| 34W10 | 21.0 ± 0.71 | 33.4 ± 0.53 | 35.6 ± 0.81 | 38.4 ± 0.11 |
| 34W12 | 31.3 ± 0.21 | 42.3 ± 0.56 | 49.3 ± 1.18 | 45.8 ± 1.01 |
| 34W14 | 28.4 ± 0.34 | 38.5 ± 0.46 | 40.0 ± 0.35 | 41.1 ± 1.01 |

**Table S3 Split Tensile Strength Data**

| **Mix** | **7 days** | **28 days** | **56 days** | **90 days** |
| --- | --- | --- | --- | --- |
| 32P10 | 2.65 ± 0.05 | 2.91 ± 0.15 | 3.45 ± 0.05 | 3.39 ± 0.15 |
| 32P12 | 2.74 ± 0.17 | 3.34 ± 0.03 | 4.01 ± 0.17 | 4.58 ± 0.07 |
| 32P14 | 3.19 ± 0.12 | 4.24 ± 0.12 | 4.34 ± 0.09 | 4.77 ± 0.12 |
| 34P10 | 2.27 ± 0.11 | 2.68 ± 0.05 | 2.62 ± 0.11 | 3.31 ± 0.05 |
| 34P12 | 2.34 ± 0.07 | 3.28 ± 0.07 | 3.32 ± 0.17 | 3.53 ± 0.07 |
| 34P14 | 2.31 ± 0.21 | 2.83 ± 0.11 | 2.99 ± 0.06 | 3.53 ± 0.12 |
| 32W10 | 2.39 ± 0.15 | 3.11 ± 0.16 | 3.44 ± 0.15 | 3.38 ± 0.06 |
| 32W12 | 2.40 ± 0.12 | 3.49 ± 0.09 | 4.01 ± 0.04 | 4.64 ± 0.08 |
| 32W14 | 3.45 ± 0.13 | 4.39 ± 0.15 | 4.46 ± 0.13 | 4.91 ± 0.07 |
| 34W10 | 2.31 ± 0.12 | 2.75 ± 0.27 | 2.81 ± 0.11 | 3.33 ± 0.04 |
| 34W12 | 2.60 ± 0.04 | 3.44 ± 0.15 | 3.81 ± 0.06 | 4.27 ± 0.15 |
| 34W14 | 2.52 ± 0.11 | 3.03 ± 0.05 | 3.41 ± 0.07 | 3.71 ± 0.19 |

**Table S4 Flexural Strength Data**

| **Mix** | **7 days** | **28 days** | **56 days** | **90 days** |
| --- | --- | --- | --- | --- |
| 32P10 | 3.75 ± 0.09 | 4.01 ± 0.18 | 5.11 ± 0.25 | 5.25 ± 0.12 |
| 32P12 | 4.51 ± 0.36 | 4.68 ± 0.29 | 5.51 ± 0.26 | 6.08 ± 0.25 |
| 32P14 | 4.75 ± 0.12 | 5.25 ± 0.21 | 6.01 ± 0.21 | 6.25 ± 0.15 |
| 34P10 | 4.01 ± 0.25 | 4.28 ± 0.25 | 5.11 ± 0.25 | 5.01 ± 0.21 |
| 34P12 | 4.13 ± 0.11 | 4.48 ± 0.31 | 5.25 ± 0.31 | 5.63 ± 0.19 |
| 34P14 | 3.25 ± 0.24 | 4.25 ± 0.17 | 5.01 ± 0.15 | 6.01 ± 0.25 |
| 32W10 | 4.01 ± 0.18 | 4.15 ± 0.18 | 5.01 ± 0.17 | 5.51 ± 0.21 |
| 32W12 | 4.38 ± 0.50 | 4.65 ± 0.31 | 5.59 ± 0.25 | 6.25 ± 0.15 |
| 32W14 | 4.51 ± 0.29 | 5.35 ± 0.21 | 6.25 ± 0.21 | 7.11 ± 0.25 |
| 34W10 | 4.11 ± 0.17 | 4.55 ± 0.15 | 5.13 ± 0.15 | 5.11 ± 0.29 |
| 34W12 | 4.23 ± 0.25 | 5.01 ± 0.41 | 5.51 ± 0.18 | 6.01 ± 0.18 |
| 34W14 | 3.51 ± 0.11 | 4.75 ± 0.21 | 4.55 ± 0.50 | 5.71 ± 0.31 |

| **ANOVA Two Way (01-03-2026 20:46:43)** | | | | | |
| --- | --- | --- | --- | --- | --- |
| **Table S5 Overall ANOVA for Potable water mixes – CS at 28 days** | | | | | |
|  | **DF** | **Sum of Squares** | **Mean Square** | **F Value** | **P Value** |
| w/b | 1 | 43.86722 | 43.86722 | 270.41438 | <0.0001 |
| Activator | 2 | 544.94778 | 272.47389 | 1679.63356 | <0.0001 |
| Interaction | 2 | 383.51444 | 191.75722 | 1182.06507 | <0.0001 |
| Model | 5 | 972.32944 | 194.46589 | 1198.76233 | <0.0001 |
| Error | 12 | 1.94667 | 0.16222 |  |  |
| Corrected Total | 17 | 974.27611 |  |  |  |
| **At the 0.05 level, the population means of w/b are significantly different.** | | | | | |
| **At the 0.05 level, the population means of Activator are significantly different.** | | | | | |
| **At the 0.05 level, the interaction between w/b and Activator is significant.** | | | | | |

| **Table S6 Overall ANOVA for Wash water mixes – CS at 28 days** | | | | | |
| --- | --- | --- | --- | --- | --- |
|  | **DF** | **Sum of Squares** | **Mean Square** | **F Value** | **P Value** |
| w/b | 1 | 1.22722 | 1.22722 | 3.82842 | 0.07407 |
| Activator | 2 | 554.96778 | 277.48389 | 865.63432 | <0.0001 |
| Interaction | 2 | 308.37444 | 154.18722 | 481 | <0.0001 |
| Model | 5 | 864.56944 | 172.91389 | 539.41941 | <0.0001 |
| Error | 12 | 3.84667 | 0.32056 |  |  |
| Corrected Total | 17 | 868.41611 |  |  |  |
| **At the 0.05 level, the population means of w/b are not significantly different.** | | | | | |
| **At the 0.05 level, the population means of Activator are significantly different.** | | | | | |
| **At the 0.05 level, the interaction between w/b and Activator is significant.** | | | | | |

| **Table S7 Overall ANOVA for Potable & Wash water mixes – CS at 28 days** | | | | | | | |
| --- | --- | --- | --- | --- | --- | --- | --- |
|  | **DF** | **Sum of Squares** | **Mean Square** | **F Value** | **P Value** | |  |
| Water | 1 | 92.16 | 92.16 | 3.72225 | 0.0632 | |  |
| Activator | 2 | 1097.20056 | 548.60028 | 22.15741 | <0.0001 | |  |
| Interaction | 2 | 2.715 | 1.3575 | 0.05483 | 0.94674 | |  |
| Model | 5 | 1192.07556 | 238.41511 | 9.62935 | <0.0001 | |  |
| Error | 30 | 742.77667 | 24.75922 |  |  | |  |
| Corrected Total | 35 | 1934.85222 |  |  |  | |  |
| **At the 0.05 level, the population means of Water are not significantly different.** | | | | | | |  |
| **At the 0.05 level, the population means of Activator are significantly different.** | | | | | | |  |
| **At the 0.05 level, the interaction between Water and Activator is not significant.** | | | | | | |  |
| **Table S8 Overall ANOVA for Potable water mixes - STS** | | | | | | | |
|  | **DF** | **Sum of Squares** | **Mean Square** | **F Value** | | **P Value** | |
| w/b | 1 | 1.445 | 1.445 | 151.3089 | | <0.0001 | |
| Activator | 2 | 1.7269 | 0.86345 | 90.41361 | | <0.0001 | |
| Interaction | 2 | 1.6219 | 0.81095 | 84.91623 | | <0.0001 | |
| Model | 5 | 4.7938 | 0.95876 | 100.39372 | | <0.0001 | |
| Error | 12 | 0.1146 | 0.00955 |  | |  | |
| Corrected Total | 17 | 4.9084 |  |  | |  | |
| **At the 0.05 level, the population means of w/b are significantly different.** | | | | | | | |
| **At the 0.05 level, the population means of Activator are significantly different.** | | | | | | | |
| **At the 0.05 level, the interaction between w/b and Activator is significant.** | | | | | | | |

| **Table S9 Overall ANOVA for Wash water mixes – STS at 28 days** | | | | | |  |
| --- | --- | --- | --- | --- | --- | --- |
|  | **DF** | **Sum of Squares** | **Mean Square** | **F Value** | **P Value** |  |
| w/b | 1 | 1.56645 | 1.56645 | 60.99091 | <0.0001 |  |
| Activator | 2 | 1.9093 | 0.95465 | 37.17002 | <0.0001 |  |
| Interaction | 2 | 1.4061 | 0.70305 | 27.37378 | <0.0001 |  |
| Model | 5 | 4.88185 | 0.97637 | 38.0157 | <0.0001 |  |
| Error | 12 | 0.3082 | 0.02568 |  |  |  |
| Corrected Total | 17 | 5.19005 |  |  |  |  |
| **At the 0.05 level, the population means of w/b are significantly different.** | | | | | |  |
| **At the 0.05 level, the population means of Activator are significantly different.** | | | | | |  |
| **At the 0.05 level, the interaction between w/b and Activator is significant.** | | | | | |  |
| **Table S10 Overall ANOVA for Potable water mixes – FS at 28 days** | | | | | | |
|  | **DF** | **Sum of Squares** | **Mean Square** | **F Value** | **P Value** | |
| w/b | 1 | 0.43245 | 0.43245 | 7.45389 | 0.01826 | |
| Activator | 2 | 1.1683 | 0.58415 | 10.06866 | 0.00271 | |
| Interaction | 2 | 1.2369 | 0.61845 | 10.65987 | 0.00218 | |
| Model | 5 | 2.83765 | 0.56753 | 9.78219 | 6.49812E-4 | |
| Error | 12 | 0.6962 | 0.05802 |  |  | |
| Corrected Total | 17 | 3.53385 |  |  |  | |
| **At the 0.05 level, the population means of w/b are significantly different.** | | | | | | |
| **At the 0.05 level, the population means of Activator are significantly different.** | | | | | | |
| **At the 0.05 level, the interaction between w/b and Activator is significant.** | | | | | | |

| **Table S11 Overall ANOVA for Wash water mixes – FS at 28 days** | | | | | |
| --- | --- | --- | --- | --- | --- |
|  | **DF** | **Sum of Squares** | **Mean Square** | **F Value** | **P Value** |
| w/b | 1 | 0.0128 | 0.0128 | 0.18856 | 0.67182 |
| Activator | 2 | 1.5376 | 0.7688 | 11.32531 | 0.00173 |
| Interaction | 2 | 0.9616 | 0.4808 | 7.08274 | 0.0093 |
| Model | 5 | 2.512 | 0.5024 | 7.40093 | 0.00222 |
| Error | 12 | 0.8146 | 0.06788 |  |  |
| Corrected Total | 17 | 3.3266 |  |  |  |
| **At the 0.05 level, the population means of w/b are not significantly different.** | | | | | |
| **At the 0.05 level, the population means of Activator are significantly different.** | | | | | |
| **At the 0.05 level, the interaction between w/b and Activator is significant.** | | | | | |

**Table S12** Sorptivity coefficients (initial and secondary) of 28-day cured concrete mixes.

| **Mix** | **Initial S_i_ (mm/s^1/2^)** | **Secondary S_s_ (mm/s^1/2^)** |
| --- | --- | --- |
| 32P10 | 8.19 x 10^-3^ | 4.23 x 10^-4^ |
| 32P12 | 8.00 x 10^-3^ | 2.84 x 10^-4^ |
| **32P14** | **6.60 x 10^-3^** | **2.72 x 10^-4^** |
| 34P10 | 8.14 x 10^-3^ | 5.82 x 10^-4^ |
| 34P12 | 1.13 x 10^-2^ | 4.55 x 10^-4^ |
| 34P14 | 8.30 x 10^-3^ | 4.42 x 10^-4^ |
| 32W10 | 7.53 x 10^-3^ | 2.87 x 10^-4^ |
| 32W12 | 5.92 x 10^-3^ | 2.30 x 10^-4^ |
| **32W14** | **6.27 x 10^-3^** | **2.13 x 10^-4^** |
| 34W10 | 7.66 x 10^-3^ | 2.44 x 10^-4^ |
| 34W12 | 7.11 x 10^-3^ | 2.14 x 10^-4^ |
| 34W14 | 7.49 x 10^-3^ | 2.50 x 10^-4^ |
| **OPC** | **6.90 x 10^-3^** | **5.76 x 10^-4^** |

**Table S13** Sorptivity coefficients (initial and secondary) of 56-day cured concrete mixes.

| **Mix** | **Initial S_i_ (mm/s^1/2^)** | **Secondary S_s_ (mm/s^1/2^)** |
| --- | --- | --- |
| 32P10 | 5.74 x 10^-3^ | 2.78 x 10^-4^ |
| 32P12 | 5.96 x 10^-3^ | 2.43 x 10^-4^ |
| **32P14** | **5.39 x 10^-3^** | **1.94 x 10^-4^** |
| 34P10 | 8.03 x 10^-3^ | 4.79 x 10^-4^ |
| 34P12 | 8.42 x 10^-3^ | 3.00 x 10^-4^ |
| 34P14 | 8.53 x 10^-3^ | 2.89 x 10^-4^ |
| 32W10 | 6.55 x 10^-3^ | 2.31 x 10^-4^ |
| 32W12 | 5.24 x 10^-3^ | 2.19 x 10^-4^ |
| **32W14** | **4.93 x 10^-3^** | **1.36 x 10^-4^** |
| 34W10 | 6.77 x 10^-3^ | 2.40 x 10^-4^ |
| 34W12 | 5.24 x 10^-3^ | 2.95 x 10^-4^ |
| 34W14 | 3.97 x 10^-3^ | 2.23 x 10^-4^ |
| **OPC** | **5.03 x 10^-3^** | **1.51 x 10^-4^** |

**Mix Design of M40-Grade Concrete Using 43-Grade OPC**

**Data:**

| Grade of concrete | = M40 |
| --- | --- |
| Characteristic strength of concrete (fck) | = 40 MPa |
| Exposure | = Severe |
| Specific Gravity of CA | = 2.65 |
| Specific Gravity of Sand | = 2.60 |
| Specific Gravity of cement | = 3.11 |
| Specific Gravity of water | = 1 |
| Water absorption of CA | = 0.45 % |
| Water absorption of Sand | = 1.02% |

**Step -1: Target strength**

| f'ck | = fck + 1.65 * S |
| --- | --- |
| Or | = 48.25 |
| f'ck | = fck + X |
|  | = 46.5 |
| Target strength | = 48.25 MPa |

**Step - 2: Air content**

| Considering air content of 1% |  |
| --- | --- |
| Vol. of air content | = 0.01 m^3^ |

**Step - 3: w/c ratio**

| From Figure 1 of IS 10262:2019 |  |
| --- | --- |
| w/c ratio | = 0.35 |

**Step - 4: Selection of water content**

| For 50mm slump, Water content | = 186 kg |
| --- | --- |
| For 100mm slump, Water content | **=** 197 kg |
| Using SP at the dosage of 1% by weight of cement leads to 23% reduction in water content | |
| Hence the water content | **=** 152 kg |

**Step - 5: Calculation of Cement content**

| Cement content | = 434 kg |
| --- | --- |
| As per IS 456:2000 Cl. 8.2.4.2, max permissible cement content is 450kg/ m^3^ | |
| Actual cement content | = 434 kg/ m^3^ |
| Actual water content | = 152 kg/ m^3^ (151.9 kg/m^3^) |
| SP content | = 4.35 kg/ m^3^ |

**Step - 6:** Proportioning of aggregate

| From Table 5 of IS 10262:2019, for 20mm downsize aggregate, for w/c 0.5, | |
| --- | --- |
| Vol. of coarse aggregate | = 0.62 |
| For w/c | = 0.35 |
| Vol. of coarse aggregate | = 0.65 |
| Vol. of fine aggregate | = 0.35 |

**Step -7:** Volume calculations

| Total volume | = 1.000 m^3^ |
| --- | --- |
| Air content | = 0.010 m^3^ |
| Volume of cement | = 0.140 m^3^ |
| Volume of water | = 0.152 m^3^ |
| Vol. of SP | = 0.004 m^3^ |
| Vol. of all in aggregate | = 0.695 m^3^ |
| Mass of CA | = 1196 kg/ m^3^ |
| Mass of sand | = 632 kg/ m^3^ |

**Step -8:** Extra water to add (dry aggregate) = 164 kg/ m^3^

**Final mix design**

| Cement | = 434 kg/ m^3^ |
| --- | --- |
| Water | = 164 kg/ m^3^ |
| Coarse aggregate | = 1196 kg/ m^3^ |
| Fine aggregate | = 632 kg/ m^3^ |
| Superplasticizer | = 4.34 kg/m³ |
